# The bifidobacterial distribution in the microbiome of captive primates reflects parvorder and feed specialization of the host

**DOI:** 10.1038/s41598-021-94824-y

**Published:** 2021-07-27

**Authors:** Nikol Modrackova, Adam Stovicek, Johanna Burtscher, Petra Bolechova, Jiri Killer, Konrad J. Domig, Vera Neuzil-Bunesova

**Affiliations:** 1 Department of Microbiology, Nutrition and Dietetics, Faculty of Agrobiology, Food and Natural Resources, Czech University of Life Sciences Prague, Kamycka 129, Prague 6, 165 00 Czechia; 2 Institute of Food Science, Department of Food Science and Technology, BOKU - University of Natural Resources and Life Sciences Vienna, Muthgasse 18, 1190 Vienna, Austria; 3 Department of Ethology and Companion Animal Science, Czech University of Life Sciences Prague, Kamycka 129, Prague 6, 165 00 Czechia; 4 Zoo Liberec, Lidove sady 425/1, Liberec 1, 460 01 Czechia; 5 Institute of Animal Physiology and Genetics V.V.I, The Czech Academy of Sciences, Videnska 1083, Prague 4, 142 20 Czechia

**Keywords:** Ecology, Evolution, Microbiology, Molecular biology, Zoology

## Abstract

Bifidobacteria, which commonly inhabit the primate gut, are beneficial contributors to host wellbeing. Anatomical differences and natural habitat allow an arrangement of primates into two main parvorders; New World monkeys (NWM) and Old World monkeys (OWM). The number of newly described bifidobacterial species is clearly elevated in NWM. This corresponds to our finding that bifidobacteria were the dominant group of cultivated gut anaerobes in NWM, while their numbers halved in OWM and were often replaced by *Clostridiaceae* with sarcina morphology. We examined an extended MALDI-TOF MS database as a potential identification tool for rapid screening of bifidobacterial distribution in captive primates. Bifidobacterial isolates of NWM were assigned mainly to species of primate origin, while OWM possessed typically multi-host bifidobacteria. Moreover, bifidobacterial counts reflected the feed specialization of captive primates decreasing from frugivore-insectivores, gummivore-insectivores, frugivore-folivores to frugivore-omnivores. Amplicon sequencing analysis supported this trend with regards to the inverse ratio of Actinobacteria and Firmicutes. In addition, a significantly higher diversity of the bacterial population in OWM was found. The evolution specialization of primates seems to be responsible for *Bifidobacterium* abundance and species occurrence. Balanced microbiota of captive primates could be supported by optimized prebiotic and probiotic stimulation based on the primate host.

## Introduction

Primates are a remarkably species-rich order of mammals^1^. Their anatomical differences and natural habitat allow their arrangement into two main parvorders. Platyrrhines, referred as New World monkeys (NWM), naturally occurring in central and southern American tropical and subtropical regions and catarrhines (Cercopithecoidea and Hominoidea), referred as Old World monkeys (OWM), coming from tropical, subtropical, and temperate regions of Asia and Africa^2^. Many primate species are endangered^3^ and they must be protected. The conservation of threatened species is a complex and demanding process consisting of elaborated breeding programs and providing of habitat sanctuaries in captive or semi-captive centres, e.g. zoological institutions or forest corridors, which usually aim to reintroduce these species back into their natural habitat^4,5^. Unfortunately, health of captive animals is compromised by emerging recurring infectious diseases mediated through human contact and habitat modifications, and frequent therapeutic doses of antibiotics^6,7^. Furthermore, captive breeding modifies primate microbiome^8,9^ and these microbial shifts can substantially affect the host’s health^10,11^. Captivity may be also associated with the occurrence of potential pathogens that further increase risk of gut dysbiosis and illnesses^12,13^.

Besides exposure to antibiotics, dietary changes and lifestyle seem to be significant modifiers of primate gut microbiome^14^. To provide nutritional needs, primates consume a wide range of plants and animal tissues and possess a variety of dietary specializations based on the proportion of individual dietary components (one type of feed component is dominating only), such as generalist feeders or omnivores, e.g. Cercopithecines^15–18^. The generalist feeders are adapted to receive a wide variety of feed components, depending on their availability in the environment, and can be split by extension into groups classified by their majority feeds, with seasonal variation in their ratio. Among the generalist feeders, there are highly frugivorous representatives, namely chimpanzees^19–21^. If there is a lack of fruit, these primates consume various feed reaching from plants, nectar, seeds to insects or small vertebrates. Such a feeding type can be described as frugivore-omnivore. If the preferred fruit is less available during the season, primates start to consume more leaves or other parts of plants. Gibbons, for instance pursue this frugivore-folivore feeding strategy^22–25^. Similarly, if the second major component alongside fruit consists of insects, primates are classified as frugivores-insectivores (tamarins)^26–30^. Exudates are another important nutritious feed apart from fruit and animal prey. Some primate species have specially adapted teeth for gum intake^31,32^. This type of feeding behaviour is called gummivory. It is typical for marmosets and can either be dominant or it can be supplemented with insect intake^33–37^. These primates are counted in the gummivore-insectivore feeding category.

Unfortunately, despite all efforts of breeders, composition of diet in captivity does not completely simulate that in the wild, in which primates consume a wider range of natural local plant and animal species^9,38^. In addition, Amato et al.^39^ points out the seasonality that is one of the natural phenomena of wild primate diet, which results in a seasonal variation of the gut microbiome.

Deviation from the natural lifestyle in captivity and associated modified diet led to a shift of native gut microbiota and a decrease in diversity and an increased relative abundance of Bacteroidetes^8,9,40,41^. Furthermore, the microbiome of captive primates displays a reduction in Actinobacteria compared to wild groups^14,41^. However, members of the *Bifidobacteriaceae* family (Actinobacteria phylum) are important natural commensals, which possess a large amount of adaptive genes involved in carbohydrate metabolism^42–44^. Moreover, bifidobacteria can utilize a diverse range of dietary carbohydrates that escape degradation in the upper parts of the intestine^45^.

Although, bifidobacterial abundance in the gut microbiota usually decreases with host aging^46^, bifidobacteria persist throughout the lifespan of primates^42,47^. Moreover, their abundance is confirmed by a recent boom of novel bifidobacterial species isolation and characterization connected to primate gut environment^48–50^.

However, data are still scarce about the bifidobacterial microbiota of captive primates and the impact of different diets. We hypothesize that the quantity and species richness of bifidobacteria in captive primates are affected by the host and feed classification. The aim of this study was to compare the quantity and diversity of bifidobacteria in faecal microbiota of captive NWM and OWM by a combination of culture-dependent and culture-independent approaches.

## Results

### Cultivation analysis

#### Quantification of cultivable bifidobacteria in primate faecal samples

**Figure 1:**
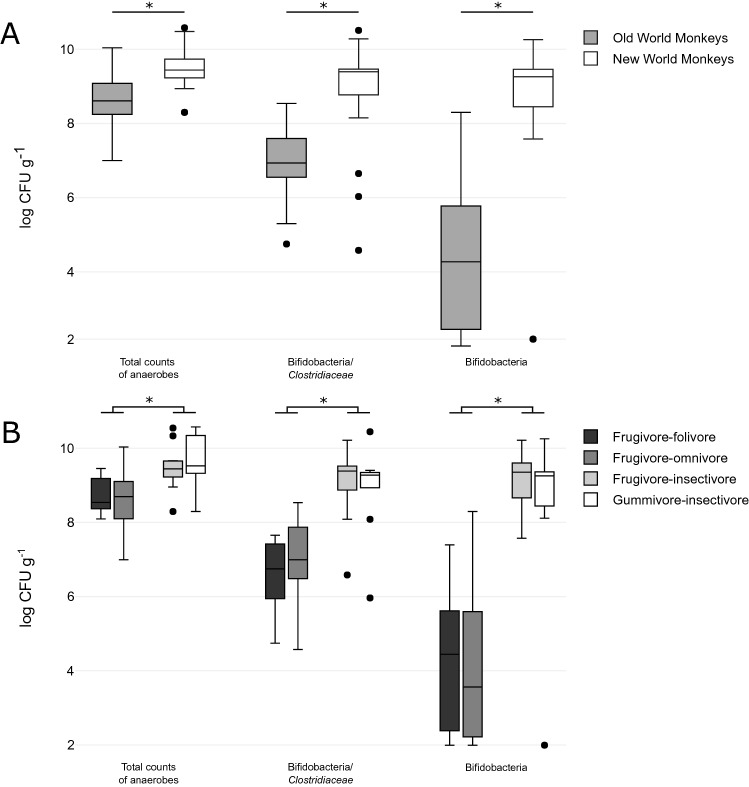
Quantification of cultivable anaerobic bacteria (log CFU g^-1^) in primate faecal samples. (**A**) Cultivation counts of bacteria per parvorder: New World monkeys (n = 24) and Old World monkeys (n = 28). (**B**) Cultivation counts of bacteria per feed category: frugivore-folivore (n = 8), frugivore-omnivore (n = 21), frugivore-insectivore (n = 13), gummivore-insectivore (n = 10). Asterisks (*) denote statistically significant differences as determined by t-test and ANOVA (p < 0.05).

Non-selective and selective media were used for the quantification of anaerobic bacteria and bifidobacteria in primate faecal samples (FS) (Table 1). Cultivation counts significantly varied between the NWM and the OWM in each monitored group of bacteria (Fig. 1A, Suppl. Tab. 1). NWM harboured significantly more anaerobic bacteria (9.52 ± 0.62 log CFU g^-1^) compared to OWM (8.62 ± 0.71 log CFU g^-1^) (t_(50)_ = 4.84, p = 1.30e-05). A similar statistically significant trend was found in colony forming units cultivated on WPS-MUP medium intended for bifidobacteria that reached 8.91 ± 1.38 log CFU g^-1^ in the NWM compared to 7.02 ± 0.93 log CFU g^-1^ in the OWM (t_(50)_ = 5.87, p = 3.50e-07). In case of FS with lower numbers of bifidobacteria and the presence of clostridia, this medium was not sufficiently selective also allowing the growth of clostridia^51,52^. Consequently, a notably greater statistically significant difference was detected between primate parvorders on more selective WSP-NORF medium with bifidobacterial counts of 8.57 ± 2.13 log CFU g^-1^ for the NWM and 4.32 ± 2.04 log CFU g^-1^ for the OWM (Z = 5.17, p = 2.38e-07). Cultivation differences between parvorders were also reflected within the primate sub-division based on feed specialization (Fig. 1B). Specifically, gummivore-insectivores (9.63 ± 0.71 log CFU g^-1^) and frugivore-insectivores (9.46 ± 0.57 log CFU g^-1^) exhibited significantly higher numbers of anaerobic bacteria including bifidobacteria than frugivore-folivores (8.72 ± 0.49 log CFU g^-1^) and frugivore-omnivores (8.60 ± 0.78 log CFU g^-1^). The same statistically significant trend was found on WPS-MUP in gummivore-insectivores (8.99 ± 1.19 log CFU g^-1^) and frugivore-insectivores (9.19 ± 0.96 log CFU g^-1^) in comparison with frugivore-folivores (6.58 ± 1.05 log CFU g^-1^) and frugivore-omnivores (7.07 ± 1.01 log CFU g^-1^), as well as on WSP-NORF in gummivore-frugivores (8.46 ± 2.34 log CFU g^-1^) and frugivore-insectivores (9.15 ± 0.76 log CFU g^-1^) compared to frugivore-folivores (4.29 ± 1.95 log CFU g^-1^) and frugivore-omnivores (4.22 ± 2.13 log CFU g^-1^) (Supplementary S5).

**Table 1:** List of monkey hosts kept in zoological gardens**.**

ID	Primate host species	Family	Parvorder	Zoo	Feed category
PR1	Common Marmoset (*Callithrix jacchus*)	*Calitrichidae*	NWM	Pilsen, CZ	Gummivore-insectivore
PR2	Common Marmoset (*Callithrix jacchus*)	*Calitrichidae*	NWM	Pilsen, CZ	Gummivore-insectivore
PR3	White-faced Saki (*Pithecia pithecia*)	*Pitheciidae*	NWM	Pilsen, CZ	Frugivore-omnivore
PR4	Emperor Tamarin (*Saguinus imperator*)	*Calitrichidae*	NWM	Pilsen, CZ	Frugivore-insectivore
PR5	Moustached Tamarin (*Saguinus mystax*)	*Calitrichidae*	NWM	Pilsen, CZ	Frugivore-insectivore
PR6	Brown-mantled Tamarin (*Saguinus fuscicollis*)	*Calitrichidae*	NWM	Pilsen, CZ	Frugivore-insectivore
PR7	Red-handed Tamarin (*Saguinus midas*)	*Calitrichidae*	NWM	Pilsen, CZ	Frugivore-insectivore
PR8	Red-handed Tamarin (*Saguinus midas*)	*Calitrichidae*	NWM	Pilsen, CZ	Frugivore-insectivore
PR9	Emperor Tamarin (*Saguinus imperator*)	*Calitrichidae*	NWM	Pilsen, CZ	Frugivore-insectivore
PR10	Silvery Marmoset (*Mico argentatus*)	*Calitrichidae*	NWM	Pilsen, CZ	Gummivore-insectivore
PR11	Silvery Marmoset (*Mico argentatus*)	*Calitrichidae*	NWM	Pilsen, CZ	Gummivore-insectivore
PR15	Silvery Marmoset (*Mico argentatus*)	*Calitrichidae*	NWM	Pilsen, CZ	Gummivore-insectivore
PR16	Emperor Tamarin (*Saguinus imperator*)	*Calitrichidae*	NWM	Pilsen, CZ	Frugivore-insectivore
PR17	Emperor Tamarin (*Saguinus imperator*)	*Calitrichidae*	NWM	Pilsen, CZ	Frugivore-insectivore
PR18	Chimpanzee (*Pan troglodytes*)	*Hominidae*	OWM	Liberec, CZ	Frugivore-omnivore
PR19	Northern White-cheeked Gibbon (*Nomascus leucogenys*)	*Hylobatidae*	OWM	Liberec, CZ	Frugivore-folivore
PR20	Golden-bellied Mangabey (*Cercocebus chrysogaster*)	*Cercopithecidae*	OWM	Liberec, CZ	Frugivore-omnivore
PR21	Diana Monkey (*Cercopithecus diana*)	*Cercopithecidae*	OWM	Liberec, CZ	Frugivore-omnivore
PR22	Lion-tailed Macaque (*Macaca silenus*)	*Cercopithecidae*	OWM	Liberec, CZ	Frugivore-omnivore
PR23	Hamadryas Baboon (*Papio hamadryas*)	*Cercopithecidae*	OWM	Liberec, CZ	Frugivore-omnivore
PR24	Pygmy Marmoset (*Cebuella pygmaea*)	*Calitrichidae*	NWM	Liberec, CZ	Gummivore-insectivore
PR26	Cotton-top Tamarin (*Saguinus oedipus*)	*Calitrichidae*	NWM	Liberec, CZ	Frugivore-insectivore
PR27	Golden Lion Tamarin (*Leontopithecus rosalia*)	*Calitrichidae*	NWM	Olomouc, CZ	Frugivore-insectivore
PR28	Common Marmoset (*Callithrix jacchus*)	*Calitrichidae*	NWM	Olomouc, CZ	Gummivore-insectivore
PR29	Patas Monkey (*Erythrocebus patas*)	*Cercopithecidae*	OWM	Olomouc, CZ	Frugivore-omnivore
PR30	Goeldi's Marmoset (*Callimico goeldii*)	*Calitrichidae*	NWM	Olomouc, CZ	Frugivore-insectivore
PR31	White-headed Marmoset (*Callithrix geoffroyi*)	*Calitrichidae*	NWM	Olomouc, CZ	Gummivore-insectivore
PR32	White-headed Marmoset (*Callithrix geoffroyi*)	*Calitrichidae*	NWM	Olomouc, CZ	Gummivore-insectivore
PR33	Moustached Tamarin (*Saguinus mystax*)	*Calitrichidae*	NWM	Olomouc, CZ	Frugivore-insectivore
PR34	Patas Monkey (*Erythrocebus patas*)	*Cercopithecidae*	OWM	Olomouc, CZ	Frugivore-omnivore
PR35	Silvery Marmoset (*Mico argentatus*)	*Calitrichidae*	NWM	Olomouc, CZ	Gummivore-insectivore
PR36	Campbell's Mona Monkey (*Cercopithecus campbelli*)	*Cercopithecidae*	OWM	Dvur Kralove, CZ	Frugivore-omnivore
PR37	Putty-nosed Monkey (*Cercopithecus nictitans*)	*Cercopithecidae*	OWM	Dvur Kralove, CZ	Frugivore-omnivore
PR38	Northern Talapoin Monkey (*Miopithecus oguensis*)	*Cercopithecidae*	OWM	Dvur Kralove, CZ	Frugivore-omnivore
PR39	De Brazza´s Monkey (*Cercopithecus neglectus*)	*Cercopithecidae*	OWM	Pilsen, CZ	Frugivore-omnivore
PR40	Northern White-cheeked Gibbon (*Nomascus leucogenys*)	*Hylobatidae*	OWM	Liberec, CZ	Frugivore-folivore
PR41	Chimpanzee (*Pan troglodytes*)	*Hominidae*	OWM	Liberec, CZ	Frugivore-omnivore
PR42	Chimpanzee (*Pan troglodytes*)	*Hominidae*	OWM	Liberec, CZ	Frugivore-omnivore
PR43	Chimpanzee (*Pan troglodytes*)	*Hominidae*	OWM	Liberec, CZ	Frugivore-omnivore
PR44	Chimpanzee (*Pan troglodytes*)	*Hominidae*	OWM	Liberec, CZ	Frugivore-omnivore
PR45	Patas Monkey (*Erythrocebus patas*)	*Cercopithecidae*	OWM	Olomouc, CZ	Frugivore-omnivore
PR46	Southern Yellow-cheeked Gibbon (*Nomascus gabriellae*)	*Hylobatidae*	OWM	Olomouc, CZ	Frugivore -folivore
PR47	Southern Yellow-cheeked Gibbon (*Nomascus gabriellae*)	*Hylobatidae*	OWM	Olomouc, CZ	Frugivore -folivore
PR51	Southern Yellow-cheeked Gibbon (*Nomascus gabriellae*)	*Hylobatidae*	OWM	Bratislava, SK	Frugivore -folivore
PR52	Green Monkey (*Chlorocebus sabaeus*)	*Cercopithecidae*	OWM	Hodonin, CZ	Frugivore-omnivore
PR55	Hamlyn’s Monkey (*Cercopithecus hamlyni*)	*Cercopithecidae*	OWM	Bojnice, SK	Frugivore-omnivore
PR56	Roloway Monkey (*Cercopithecus roloway*)	*Cercopithecidae*	OWM	Bojnice, SK	Frugivore-omnivore
PR57	Lesser Spot-nosed Monkey (*Cercopithecus petaurista*)	*Cercopithecidae*	OWM	Bojnice, SK	Frugivore-omnivore
PR58	Southern Yellow-cheeked Gibbon (*Nomascus gabriellae*)	*Hylobatidae*	OWM	Bojnice, SK	Frugivore-folivore
PR59	Northern White-cheeked Gibbon (*Nomascus leucogenys*)	*Hylobatidae*	OWM	Liberec, CZ	Frugivore-folivore
PR60	Northern White-cheeked Gibbon (*Nomascus leucogenys*)	*Hylobatidae*	OWM	Liberec, CZ	Frugivore-folivore
PR61	Golden Lion Tamarin (*Leontopithecus rosalia*)	*Calitrichidae*	NWM	Olomouc, CZ	Frugivore-insectivore

General information about primate taxonomy, parvorder and feed classification.

Primate general feeders (n = 52) were grouped to 4 individual feed categories based on proportion of dominating feed components – frugivore-omnivore, frugivore-folivore, frugivore-insectivore, and gummivore-insectivore. Zoo, zoological garden; CZ, Czechia; SK, Slovakia; NWM, New World monkey; OWM, Old World monkey.

#### Bifidobacterial species detected by MALDI-TOF MS

**Figure 2:**
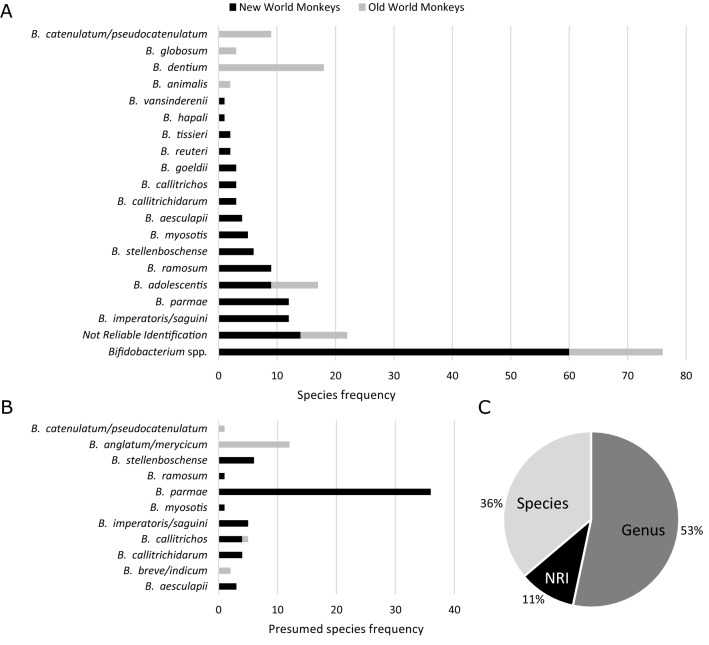
MALDI-TOF MS identification of primate bifidobacterial isolates. (**A)** MALDI-TOF MS identification of 210 bifidobacterial strains. (**B)** The closest probable species match of isolates with unambiguous genus MALDI-TOF MS identification (*Bifidobacterium* spp). (**C)** Proportion of species assignment, genus assignment and not reliable identification (NRI) of bifidobacterial isolates. Bruker criteria (scores) for assignment: 0.000–1.699 not reliable identification, 1.700–1.999 probable genus identification, 2.000–3.000 genus and species identification.

Bacterial colonies with variable cultivation characteristics from bifidobacterial selective media were isolated for further identifications (Suppl. Tab. 1). From a total of 326 isolates, 210 were F6PPK-positive bifidobacteria and the remaining 116 isolates (isolated mainly from WSP-MUP) were F6PPK-negative gas producing clostridial rods or cells with sarcina morphology. All F6PPK-positive strains were also identified with MALDI-TOF MS using an expanded custom database for bifidobacterial identification. 54% of the strains (n = 112) were assigned to 18 different bifidobacterial species, 36% (n = 76) were assigned only to the *Bifidobacterium* genus, and 11% (n = 22) were not identified reliably (Fig. 2A, C).

*B. parmae, B. imperatoris/saguini,* and *B. ramosum* were the most frequently identified species in the NWM, whereas *B. dentium* and *B. catenulatum/pseudocatenulatum* were most common in the OWM. Interestingly, *B. adolescentis* was equally represented in both primate parvorders. A more diverse species representation of bifidobacteria was found in the NWM (14 spp.) compared to the OWM (5 spp.). Genus-level assignment and the presence of not reliable identifications (NRI) was mainly detected in the NWM. Related presumed species compliance and the closest match of *Bifidobacterium* spp. strains was found predominantly with *B. parmae* and *B. stellenboschense* in the NWM, and *B. angulatum/merycicum* in the OWM (Fig. 2B).

#### Species assignment verification by 16S rRNA gene sequencing

The MALDI-TOF MS identification was verified by 16S rRNA gene Sanger sequencing of 46 strains, whose selection was randomly executed based on determined species frequency and identification scores (Suppl. Tab. 2). Due to similar MALDI-TOF MS spectra, some bifidobacterial species could not be distinguished. However, the results consistently suggest an assignment to either of the two indistinguishable species. These indistinguishable groups were merged to produce consistent MALDI-TOF MS assignment and are presented together in the following groups: *B. angulatum/merycicum*, *B. breve/indicum*, *B. catenulatum/pseudocatenulatum*, and *B. imperatoris/saguini*.

An agreement between the MALDI-TOF MS species assignment and the sequencing of 16S rRNA gene was confirmed for 38 strains. Only 3 strains were identified differently by the two methods. Namely, strain N127 identified as *B. faecale* by 16S rRNA gene sequencing was mistaken for *B. adolescentis* by the MALDI-TOF MS, *B. imperatoris* for NRI (N40), and PEBJ_s for *B. imperatoris/saguini* (N50). Interestingly, mentioned strain N50 together with N74, N94, N97, and N115, exhibiting MALDI-TOF MS NRI score (< 1.69), were considered potential novel species of bifidobacteria. In addition, this sample set also contained 5 problematic strains (N16, N70, N81, N119, and N125), whose 16S rRNA gene sequencing failed repeatedly and thus their MALDI-TOF MS identity was not confirmed.

### Amplicon sequencing analysis

**Figure 3:**
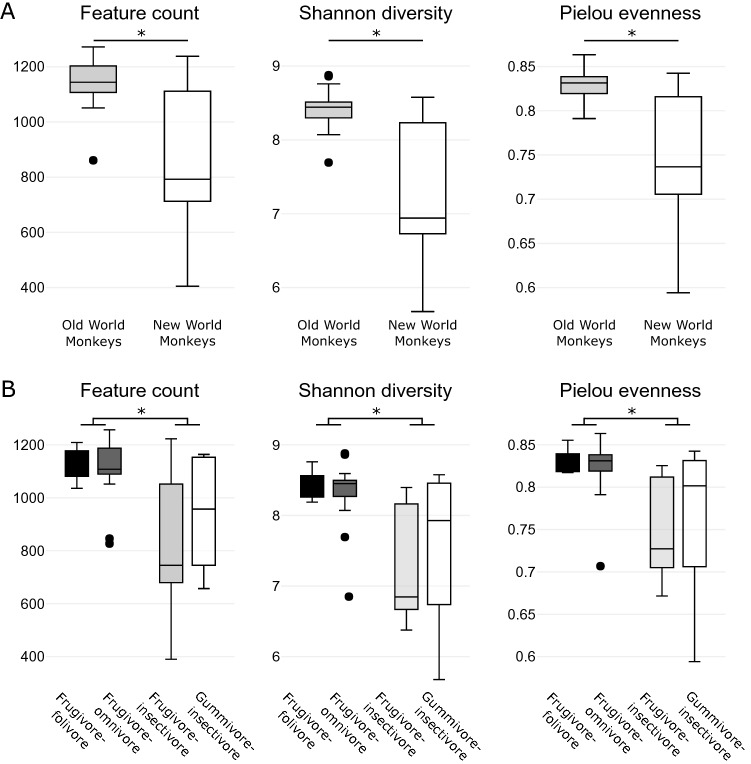
Alfa-diversity of primate gut microbiota. (**A**) Bacterial α-diversity per parvorder: New World monkeys and Old World monkeys. (**B**) Bacterial α-diversity per feed category: frugivore-folivore, frugivore-omnivore, frugivore-insectivore, gummivore-insectivore. Asterisks (*) denote adjusted statistically significant differences (adj. p < 0.05).

Amplicon sequencing profiles of the FS collected from captive primates were determined by sequencing the V4 region of the 16S rRNA gene. The bacterial α-diversity was expressed as an ASV count, Shannon diversity, and Pielou evenness. Each diversity parameter between the primate parvorders was significantly higher in the OWM (ASV count: F_(1,50)_ = 30.47, p = 1.21 × 10^–6^, η^2^ = 0.379, Shannon: F_(1,50)_ = 38.01, p = 1.21e-07, η^2^ = 0.432, Pielou: F_(1,50)_ = 38.41, p = 1.08e-07, η^2^ = 0.434) (Fig. 3A). Similarly, there was a significantly higher diversity, evenness, and richness of the bacterial population in the frugivore-folivores and frugivore-omnivores compared to the frugivore-insectivores and gummivore-insectivores (Fig. 3B, Supplementary S1).

**Figure 4:**
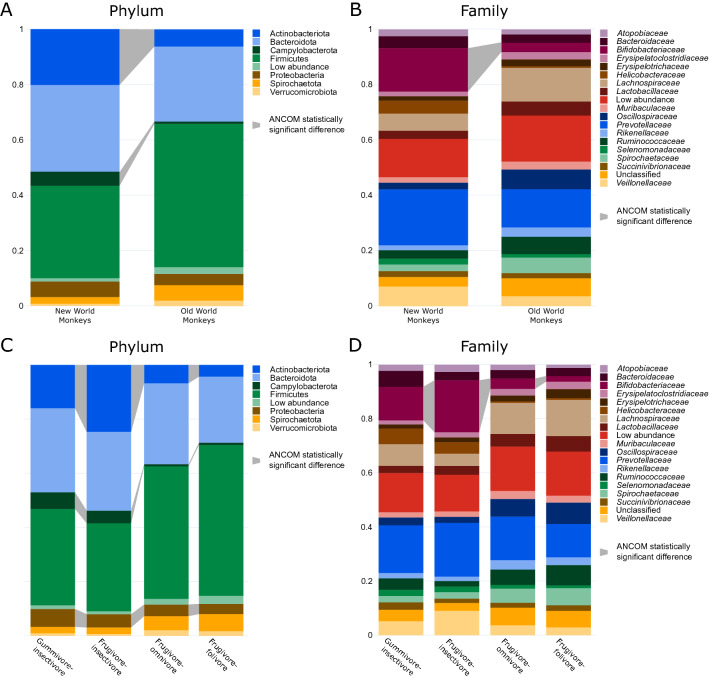
Relative abundance of primate gut microbiota. (**A**) Relative abundance of bacteria within parvorders on phylum level. (**B**) Relative abundance of bacteria within parvorders on family level. (**C**) Relative abundance of bacteria within feed categories on phylum level. (**D**) Relative abundance of bacteria within feed categories on family level. ANCOM statistically significant differences are denoted with grey links.

Microbial community shifts were found between the NWM and OWM parvorders. The relative abundance of phylum Actinobacteriota (W = 13) and Campylobacterota (W = 12) was significantly higher in the NWM compared to the OWM as confirmed by the ANCOM statistics. Meanwhile, the phylum Firmicutes showed an opposite trend, which was however not statistically significant (Fig. 4A). The difference in the Actinobacteriota can be attributed specifically to the family *Bifidobacteriaceae* which was significantly higher in the NWM (16%) compared to the OWM (3%) (W = 139) (Fig. 4B, Supplementary S2). These findings corroborate the cultivation results.

The proportion of the phylum Actinobacteriota was statistically significantly different among the primate feed categories (W = 12) and it was the highest in the frugivore-insectivores followed by gummivore-insectivores, frugivore-omnivores, and frugivore-folivores. Furthermore, the phyla Proteobacteria and Campylobacterota were statistically significantly different among the categories (W = 8, W = 7 respectively) with a notable enrichment of both in the frugivore-insectivores followed by gummivore-insectivores compared to frugivore-omnivores and frugivore-folivores. Moreover, although not statistically significant, the opposite ratio of Firmicutes was also detected (Fig. 4C). The relative abundance of *Bifidobacteriaceae* was significantly different across the categories (W = 140); the most abundant in the frugivore-insectivores (19%), followed by the gummivore-insectivores (12%), the frugivore-omnivores (4%), and the frugivore-folivores (2%) (Fig. 4D).

By comparing 16S rRNA gene sequencing data of cultured bifidobacterial isolates with the results of 16S rRNA gene amplicon sequencing of the FS, we retrospectively confirmed the presence of 18 species within this sample set. *B. callitrichos* and *B. parmae* were significantly enriched in the NWM (W = 38, W = 38 respectively), followed by *B. saguini* (W = 34)*, B. biavatii* (W = 34), *B. vansinderenii* (W = 34)*, B. aerophilum* (W = 34), unclassified II ASV (W = 33) and sp. I ASV (W = 30). The distribution of bifidobacteria corresponds to the proportion of *Bifidobacteriaceae* among the total relative bacteria in samples normalized to 42 134 sequences/sample in the primate feed categories as determined by amplicon sequencing.

## Discussion

Dynamic microbial communities aid the living and surviving of animals in changing environmental conditions, including habitat degradation, captive breeding, and diet. If microbial balance of the host is disturbed and dysbiosis occurs, there is a presumption of disease development^5,53,54^. Among others, commensal microorganisms, such as bifidobacteria, play a crucial role in maintaining the gut homeostasis^55–57^. Bifidobacterial diversity and adaptation are connected to their hosts and environments with possession of specific genomic traits^58–60^ which includes primates^42^.

Two independent approaches, cultivation with subsequent MALDI-TOF MS identification and amplicon sequencing of the V4 region of the 16S rRNA gene, were used to analyse the microbiome composition and the prevalence of bifidobacterial species in primate gut microbiota. NWM are a significant source of cultivable bifidobacteria with average counts of 10^8^ CFU g^-1^ of faeces compared to the OWM with four orders of magnitude lower counts. Interestingly, although no health complications were evident, FS of primate individuals with reduced or undetectable cultivation counts of bifidobacteria contained *Clostridiaceae*, mainly displaying sarcina morphology. This was mainly observed in individuals belonging to the OWM parvorder (Suppl. Tab. 1). Spore-forming bacteria identified as *Sarcina ventriculi* (syn. *Clostridium ventriculi*) were previously isolated also from primates without apparent health problems^61–63^. Although they are considered pathogens^64^, this may indicate sarcina as common bacteria of the primate gut microbiota. In the gut of NWM, the abundance of sarcina is probably decreased by the presence of bifidobacteria, which exhibit potential to hamper growth of clostridia^65–67^. The inverse ratio and balancing of the bifidobacteria and clostridia are typically described in the gut microbiome of infants^68–70^.

Timperio et al.^71^ showed that the screening of bacterial isolates from environmental samples can be performed efficiently, quickly, and inexpensively using MALDI-TOF MS and should be refined by implementation of environmental strains into the database. Within our study, the use of an extended custom database for MALDI-TOF MS allowed reliable species differentiation and identification of wild bifidobacterial isolates. Higher species diversity was observed in NWM. Interestingly, the multi-host species *B. adolescentis* was present among most screened captive primates. In OWM *B. dentium* and *B. catenulum/pseudocatenulatum*, that are common species of the human gut microbiota, as well as *B. adolescentis,* were found^72^. Lugli et al.^42^ detected *B. adolescentis* and *B. dentium* in OWM as well, and indicated possible joint development and evolutionary relatedness. In contrast, NWM exhibited the presence of cultivable bifidobacteria mainly with primate origin. Interestingly, Brown et al.^73^ pointed out that marmoset bifidobacteria are closely related to those in tamarins. Furthermore, we found that bifidobacterial species variability in NWM significantly exceeds that in OWM. Furthermore, we hereby confirmed that we can re-isolate recently described primate *Bifidobacterium* spp. also from primate species with various captive locations other than those from which bifidobacteria were originally isolated.

Moreover, MALDI-TOF MS screening allowed us to identify 5 potential novel species of bifidobacteria isolated from tamarins that were confirmed by 16S rRNA gene sequencing. That indicates primate gut as a promising environment for the discovery of novel species of bifidobacteria^42,48,50^. To achieve an accurate identification of potential novel species, a combination with other methods, such as sequencing of phylogenetic markers^74–76^, multi-locus sequence typing^77^, and genome sequencing^78^, should be included.

The significantly lower species richness and high relative abundance of bifidobacteria in NWM compared to OWM was confirmed by sequencing of the V4 region of 16S rRNA gene. The relative abundance of *Bifidobacteriaceae* reached 16% in the NWM and only 3% in the OWM. The same trend was also detected for *Prevotellaceae* and *Veillonellaceae*. In particular, marmosets and tamarins exhibited 32% bifidobacterial abundance compared to 0.03% in the OWM^42^. This high relative bifidobacterial proportion in adult marmosets could be a consequence of their housing as family groups and their constant subjection to the gut microbiota of other individuals^73^. Conversely, *Lachnospiraceae*, *Oscillospiraceae*, *Ruminococcaceae*, and *Spirochaetaceae* showed an opposite trend with high abundances in OWM. Interestingly, we showed that the captive NWM have high relative levels of bifidobacteria, which is similar to what they display in the wild^47,79–81^. It indicates that NWM gut is a rich bifidobacterial environment that is also supported by other studies^42,82,83^. In contrast to our results in captive individuals, some microbiome studies point to a slightly increased bifidobacterial relative proportions in wild OWM as well^84,85^. Although the captivity was previously described as a factor influencing the presence of Actinobacteria in the primate gut microbiome^14,41^, our results suggest that it is probably not as strong as the affiliation to the primate parvorder, which seems to be considerably more significant.

Primate gut microbiome seems to be significantly modified by dietary changes of the host species and geography^14^. Frugivore-insectivores and gummivore-insectivores possessed significantly more abundant *Bifidobacteriaceae* compared to frugivore-omnivores and frugivore-folivores. Interestingly, if insects constitute an important component of the diet, bifidobacteria are highly abundant. Ecologically beneficial symbionts leading to host evolutionary dependence have been previously described in other animal taxa, such as sap-feeding insects, which generate essential amino acids exclusively for their microbial symbionts^86^. Bifidobacteria are known as a commensal bacterial group of insects with social life^87^, whereas the importance of insects in the diet of primates in relation to bifidobacterial occurrence remains unclear.

Although captive feeding inevitably modifies primate gut microbiome to decreased diversity, the feed optimization could improve the animals health condition^40^. In contrast to Amato et al.^88^, who state that the host phylogeny is stronger driver in shifts of microbial composition than the diet and geographic location, our results suggest that both diet and the host itself affect the microbiome composition, especially the relative abundance of *Bifidobacteriaceae*. Moreover, it is important to mention, that the diet of captive animals usually includes fruits, vegetables, and leaves that may not completely match the available components present in the wild. In addition, the natural microbiota reflects diet seasonality and location that may affect trophic interactions in the gastrointestinal tract of the host^89,90^.

Clayton et al.^91^ confirms that modified diet in captive primates is related to the alteration of microbiome composition and host health. Captive primate individuals susceptible to health disorders may show clinical signs including chronic diarrhoea, weight loss, lethargy, cardiac disease, and poor reproductive success^9,12,92,93^. Therefore, it is necessary to further monitor the relationship between the microbiome, diet, and the health of captive primates^40^. Microbiota modulation is an effective and affordable strategy for host health support of threatened animals^5^. Therefore, applicable mitigation strategies such as optimized dietary^40^ and prebiotic interventions^94^ could be pursued towards supporting balanced microbiota in captive primates. Moreover, probiotic supplementation with focus on bifidobacteria, that naturally colonize primate guts, can be a further promising approach^42,43,95^. Furthermore, this may provide a potential approach in human probiotic intervention. Due to the ever-decreasing diversity of the human microbiome through diet and antimicrobial intake, the microbiome of originally living evolutionarily close relatives has the potential to design a probiotic that is no longer part of the human microbiota and could have the potential to strengthen health^96^. Probiotic intervention should be optimized according to the gut microbiota composition and should be supported by appropriately selected prebiotic stimulation in synbiotic mixtures for long-term maintenance of balanced microbiome and host health.

## Materials and methods

### Sampling and cultivation analysis

Faecal samples of primate hosts (n = 52) belonging to two parvorders, NWM (n = 24) and OWM (n = 28), were preliminary screened for quantitative content of cultivable bifidobacteria. The list of primate hosts and classification into parvorders and feed category is shown in Table 1. Sampling was performed in zoological gardens in Dvur Kralove, Hodonin, Liberec, Olomouc, Pilsen (all Czechia), Bojnice, and Bratislava (both Slovakia) between 2017–2019. FS were collected in tubes containing dilution buffer (5 g L^-1^ tryptone, 5 g L^-1^ nutrient broth No. 2, 2.5 g L^-1^ yeast extract (all Oxoid, Basingstoke, UK), 0.5 g L^-1^ L-cysteine, 1 mL L^-1^ Tween 80 (both Sigma-Aldrich, St. Louis, Missouri, USA), 30% glycerol (VWR, Radnor, Pennsylvania, USA), and glass pearls for homogenization. Media were prepared in an oxygen‐free carbon dioxide environment^97^ and then sterilized. After sampling, the tubes were stored at –20 °C and within the 14 days transported into the laboratory for analysis. Then, decimal serial dilutions of FS were spread on the following media.

Wilkins-Chalgren Anaerobe Agar was supplemented with 5 g L^-1^ GMO-Free Soya Peptone (both Oxoid), 0.5 g L^-1^ L-cysteine, and 1 mL L^-1^ Tween 80 to determine total counts of anaerobic bacteria (WSP medium). Moreover, two selective media were used for bifidobacterial quantification and isolation: WSP-NORF (WSP agar supplemented with 100 mg L^-1^ of mupirocin, 200 mg L^-1^ of norfloxacin (both Oxoid), and 1 mL L^-1^ of acetic acid (Sigma-Aldrich)^52^) and WSP-MUP (WSP agar supplemented with 100 mg L^-1^ of mupirocin and 1 mL L^-1^ of acetic acid^98^). All plates were incubated anaerobically using GENbag anaer (bioMérieux, Craponne, France) at 37 °C for 2 days.

### Isolation and culture identifications

Based on variable cultivation characteristics, the isolation of colonies from selective media and consecutive sub-cultivation was performed in tubes containing WSP broth under anaerobic conditions^97^ at 37 °C for 1 day. Whether a culture belonged to *Bifidobacterium* spp. was verified by fructose-6-phosphate phosphoketolase (F6PPK) test with cetrimonium bromide for cell disruption according to Orban and Patterson (2000)^99^. Subsequently, bifidobacterial isolates were identified to the species level using Matrix-Assisted Laser Desorption/Ionization Mass Spectrometry (MALDI-TOF MS) with ethanol-formic acid extraction procedure with HCCA matrix solution according to the manufacturer’s instructions (Bruker Daltonik GmbH, Bremen, Germany). An extended custom database (based on Bruker Biotyper software tools), which included 50 additional bifidobacterial species in addition to the already available entries, was used for identification. An overview about the database entries is provided in Suppl. Tab. 3. Stock cultures of bifidobacteria were stored at –80 °C in 30% glycerol.

Selected isolates (n = 46) were further identified by 16S rRNA gene amplicon sequencing. DNA was isolated from freshly grown bifidobacterial cultures in WSP broth using PrepMan Ultra™ (Applied Biosystems, Waltham, Massachusetts, USA) according to manufacturer's instructions and stored at –20 °C. Primers 285F (5′-GAGGGTTCGATTCTGGCTCAG-3′) and 261R (5′-AAGGAGGTGATCCAGCCGCA-3′) were used for PCR amplification of nearly the full 16S rRNA gene according to Kim et al.^100^ enabling longer reads and thus more precise taxonomic identification. PCR products were purified using the E.Z.N.A. Cycle Pure Kit (Omega Bio-Tek, Norcross, Georgia, USA) and sequenced by Eurofins Genomics (Ebersberg, Germany). The obtained sequences were processed in Chromas Lite 2.5.1 (Technelysium Pty Ltd., Tewantin, Australia), BioEdit^101^ with ClustalW algorithm^102^, and compared with 16S rRNA gene sequences in BLAST rRNA/ITS (https://blast.ncbi.nlm.nih.gov/) and EZBioCloud databases (https://www.ezbiocloud.net/). The sequences of the 16S rRNA gene are available in the GenBank database under accession numbers MN736337–341, 342, 344–346, 348, 350–355, 357–360, 363–365, 367, 369, 372–378, 381, 387–388, 390–392, and MW678772–74.

### Amplicon sequencing analysis

Total genomic DNA was extracted from 200 mg of FS using the Fast DNA SPIN kit for soil (MP Biomedicals, Illkirch-Graffenstaden, France) according to the manufacturer's instructions. The DNA concentration of each sample was determined using the Qubit 1X dsDNA HS Assay Kit (Invitrogen, Paisley, UK) and a Qubit fluorometer. Subsequent library preparation and sequencing were performed by NovoGene (Cambridge, UK). As amplicon sequencing method supports only shorter fragments, the V4 region of the 16S rRNA gene (300 bp fragments) was amplified using primers 515F (5′-GTGCCAGCMGCCGCGGTAA-3′) and 806R (5′-GGACTACHVGGGTWTCTAAT-3′) and a Phusion High-Fidelity PCR Master Mix (New England Biolabs, Ipswich, Massachusetts, USA). The library was prepared using the NEB Next® UltraTM DNA Library Prep Kit for Illumina and paired-end 250 bp sequencing was performed using the NovaSeq machine (Illumina, San Diego, California, USA). The resulting sequences were submitted to the NCBI database with the accession number ERP128111. Amplicon sequence variants (ASV) were obtained using the DADA2 pipeline (bioconductor-dada2 v1.16.0)^103^ and Silva non redundant database v138^104^ (Supplementary S3) with custom manual species assignment. The depth of sequencing of the resulting data was normalized by rarefaction to the lowest sequencing depth (42 134 sequences/sample) and a relative abundance on several taxonomic levels in different variable groups were explored (Supplementary S4). Total bacterial diversity was expressed as Shannon entropy^105^, the population richness was expressed as simple feature or ASV counts and the evenness was expressed as Pielou’s index^106^.

### Statistical analyses

Counts of bacterial colonies in log CFU g^-1^ within the parvorders and feed categories are shown as boxplots. The normality of data was evaluated by Shapiro–Wilk W test (α = 0.05). Differences in bacterial counts were assessed using a Mann–Whitney U Test (α = 0.05) within the parvorders, and a one-way ANOVA within the feed categories (α = 0.05) using STATISTICA software (StatSoft, Prague, Czechia) and Microsoft Office Professional Plus 2016.

To detect differentially abundant taxa between the sample categories, the ANCOM statistical test^107^ was used from the package skbio v0.5.2 (scikit-bio.org). The one-way F statistics from the scipy package v1.4.1^108^ was used to determine that statistical significance with α = 0.05. Several categories of the data were explored on both the Phylum and Family level. Furthermore, the bifidobacterial sub-population was extracted for each sample and the differentially abundant species were calculated. Statistically significant results are presented in form of boxplots (Supplementary S2).

The statistical significance of difference in means of the diversity metrics (Shannon, Pielou, and ASV counts) was assessed using the ordinary least squares method coupled with a pairwise T-test. The data was Box-Cox transformed and the resulting residuals were normally distributed (Jarque-Berra and Omnibus probability > 0.05), however, the groups were highly heteroskedastic. To mitigate this, we have used the ordinary least square method from the package statsmodels v0.11.0^109^ with MacKinnon and White’s heteroscedasticity robust standard errors^110^ (Supplementary S1).

### Ethical approval

The sampling of primate faeces was performed during routine daily procedures. All procedures involving animals adhered to recommendations of the “Guide for the Care and Use of Animals” by the Czech University of Life Sciences Prague. The research conducted herein was approved by Ethic and Animal Care Committee of the Czech University of Life Sciences Prague (protocol number: CZU/17/19) and was performed in accordance with the relevant guidelines and regulations. All zoological institutions have rigorous standards for animal welfare and are accredited by the European Association of Zoos and Aquaria. The research adhered to the legal requirements of the Czech Republic for the ethical treatment of nonhuman primates as well as in accordance with European Directive 2010/63/EU.

## Supplementary Information

Supplementary files.
