# Supplementary material for: The bifidobacterial distribution in the microbiome of captive primates reflects parvorder and feed specialization of the host
Source: Sci Rep. 2021 Jul 27;11:15273. doi: 10.1038/s41598-021-94824-y (PMC8316555; doi:10.1038/s41598-021-94824-y)
Supplement: Supplementary file 1 — Supplementary files. [file 41598_2021_94824_MOESM1_ESM.pdf]

Supplementary information

The bifidobacterial distribution in the microbiome of captive primates reflects parvorder and feed specialization of the host

Nikol Modrackova[1], Adam Stovicek[1], Johanna Burtscher[2], Petra Bolechova[3,4], Jiri Killer[1,5], Konrad J. Domig[2], and Vera Neuzil-Bunesova[1,*]

[1] Department of Microbiology, Nutrition and Dietetics, Czech University of Life Sciences Prague, Kamycka 129, Prague 6, 165 00, Czechia; [2] Institute of Food Science, Department of Food Science and Technology, BOKU - University of Natural Resources and Life Sciences Vienna, Muthgasse 18, 1190 Vienna, Austria; [3] Department of Ethology and Companion Animal Science, Czech University of Life Sciences Prague, Kamycka 129, Prague 6, 165 00, Czechia; [4] Zoo Liberec, Lidove sady 425/1, Liberec 1, 460 01, Czechia; [5] Institute of Animal Physiology and Genetics v.v.i., the Czech Academy of Sciences, Videnska 1083, Prague 4, 142 20, Czechia; [*]**Corresponding author** – Vera Neuzil-Bunesova, Department of Microbiology, Nutrition and Dietetics, Faculty of Agrobiology, Food and Natural Resources, Czech University of Life Sciences Prague, Kamycka 129, Prague 6, 165 00, Czechia. E-mail: bunesova@af.czu.cz

This file includes:

- **Supplementary Table 1** – Cultivation bacterial counts (log CFU g^{-1}) with detected isolates after microbiological analysis of primate faecal samples
- **Supplementary Table 2** – Species MALDI-TOF MS assignment verification by 16S rRNA sequencing
- **Supplementary Table 3** – List of BRUKER and new database entries (MSPs) for bifidobacterial MALDI-TOF MS identification
- **Supplementary S1** – Diversity statistics
- **Supplementary S2** – ANCOM statistics
- **Supplementary S3** – DADA2 analysis
- **Supplementary S4** – Sequence analysis
- **Supplementary S5** – ANOVA

Supplementary Table 1. Cultivation bacterial counts (log CFU g^{-1}) with detected isolates after microbiological analysis of primate faecal samples

ID	Primate host PARVORDER (FEED CATEGORY)	Cultivation analysis (log CFU g^{-1})			Bacterial isolates	Identified species by MALDI-TOF MS
		WSP	MUP	NORF		
PR1	Common Marmoset (*Callithrix jacchus*) NWM (G-I)	9,33	9,39	9,32	Bif (6)	*B. myosotis*, *B. parmae*, NRI
PR2	Common Marmoset (*Callithrix jacchus*) NWM (G-I)	9,63	6,03	2,00	Cl (6)	NRI^{S}
PR3	White-faced Saki (*Pithecia pithecia*) NWM (F-O)	9,20	4,58	2,00	Cl (5)	*C. baratii*
PR4	Emperor Tamarin (*Saguinus imperator*) NWM (F-I)	8,96	8,54	8,45	Bif (8)	*B. parmae*
PR5	Moustached Tamarin (*Saguinus mystax*) NWM (F-I)	10,55	10,24	10,22	Bif (6)	*B. adolescentis*, *B. parmae*, *B. vansinderenii*, NRI
PR6	Brown-mantled Tamarin (*Saguinus fuscicollis*) NWM (F-I)	9,29	9,37	9,14	Bif (7)	*B. adolescentis*, *B. goeldii*, *B. imperatoris/saguini*, *B. parmae*
PR7	Red-handed Tamarin (*Saguinus midas*) NWM (F-I)	9,63	9,45	9,52	Bif (10)	*B. parmae*, *B. stellenboschense*, NRI
PR8	Red-handed Tamarin (*Saguinus midas*) NWM (F-I)	9,46	9,46	9,36	Bif (7)	*B. imperatoris/saguini*, *B. parmae*, *B. stellenboschense*, NRI
PR9	Emperor Tamarin (*Saguinus imperator*) NWM (F-I)	9,45	9,40	9,40	Bif (4)	*B. parmae*, *B. stellenboschense*, NRI
PR10	Silvery Marmoset (*Mico argentatus*) NWM (G-I)	10,48	9,40	9,17	Bif (5)	*B. imperatoris/saguini*, *B. parmae*
PR11	Silvery Marmoset (*Mico argentatus*) NWM (G-I)	10,35	9,29	9,20	Bif (9)	*B. imperatoris/saguini*, *B. goeldii*, *B. parmae*
PR15	Silvery Marmoset (*Mico argentatus*) NWM (G-I)	8,30	8,15	8,12	Bif (7)	*B. imperatoris/saguini*, *B. parmae*, *B. ramosum*, NRI
PR16	Emperor Tamarin (*Saguinus imperator*) NWM (F-I)	9,67	9,87	9,75	Bif (4)	*B. parmae*
PR17	Emperor Tamarin (*Saguinus imperator*) NWM (F-I)	8,30	8,15	8,12	Bif (6)	*B. parmae*, *B. ramosum*, *B. stellenboschense*, NRI
PR18	Chimpanzee (*Pan troglodytes*) OWM (F-O)	8,89	8,54	8,30	Bif (6)	*B. cate/pseudocatenulatum*, *B. dentium*
PR19	Northern White-cheeked Gibbon (*Nomascus leucogenys*) OWM (F-F)	9,46	7,48	7,40	Bif (7)	*B. cate/pseudocatenulatum*
PR20	Golden-bellied Mangabey (*Cercocebus chrysogaster*) OWM (F-O)	8,78	6,46	6,50	Bif (5)	*B. adolescentis*, *B. angulatum/merycicum*, NRI
PR21	Diana Monkey (*Cercopithecus diana*) OWM (F-O)	10,04	6,50	6,24	Bif (5)	*B. angulatum/merycicum*, *B. breve/indicum*, NRI
PR22	Lion-tailed Macaque (*Macaca silenus*) OWM (F-O)	9,22	7,53	5,39	Bif (5)	*B. angulatum/merycicum*, *B. breve/indicum*, *B. callitrichos*, *B. dentium*
PR23	Hamadryas Baboon (*Papio hamadryas*) OWM (F-O)	9,34	6,76	5,38	Bif (9)	*B. angulatum/merycicum*, *B. adolescentis*, *B. cate/pseudocatenulatum*, *B. dentium*
PR24	Pygmy Marmoset (*Cebuella pygmaea*) NWM (G-I)	8,94	9,00	8,45	Bif (9)	*B. aesculapii*, *B. parmae*, , *B. ramosum*, *B. reuteri*
PR26	Cotton-top Tamarin (*Saguinus oedipus*) NWM (F-I)	9,14	6,65	7,58	Bif (6)	*B. callitrichidarum*, NRI
PR27	Golden Lion Tamarin (*Leontopithecus rosalia*) NWM (F-I)	10,34	10,28	10,07	Bif (7)	*B. callitrichidarum*, *B. imperatoris/saguini*, *B. callitrichos*, *B. parmae*, NRI
PR28	Common Marmoset (*Callithrix jacchus*) NWM (G-I)	9,42	9,28	9,33	Bif (5)	*B. aesculapii*, *B. callitrichos*, *B. hapali*, *B. myosotis*
PR29	Patas Monkey (*Erythrocebus patas*) OWM (F-O)	8,00	6,33	2,60	Bif (1), Cl (6)	*B. adolescentis*, NRI^{S}
PR30	Goeldi's Marmoset (*Callimico goeldii*) NWM (F-I)	9,26	9,07	8,74	Bif (3)	*B. adolescentis*, *B. callitrichidarum*
PR31	White-headed Marmoset (*Callithrix geoffroyi*) NWM (G-I)	9,80	9,47	9,39	Bif (5)	*B. aesculapii*, *B. callitrichos*, NRI
PR32	White-headed Marmoset (*Callithrix geoffroyi*) NWM (G-I)	9,43	9,41	9,37	Bif (6)	*B. aesculapii*, *B. callitrichos*, *B. myosotis*, NRI
PR33	Moustached Tamarin (*Saguinus mystax*) NWM (F-I)	9,66	9,49	9,56	Bif (16)	*B. adolescentis*, *B. imperatoris/saguini*, *B. parmae*, *B. stellenboschense*, NRI
PR34	Patas Monkey (*Erythrocebus patas*) OWM (F-O)	8,00	7,80	7,93	Bif (3)	*B. angulatum/merycicum*, NRI
PR35	Silvery Marmoset (*Mico argentatus*) NWM (G-I)	10,58	10,51	10,26	Bif (5)	*B. adolescentis*, *B. callitrichos*, *B. ramosum*, *B. tissieri*
PR36	Campbell's Mona Monkey (*Cercopithecus campbelli*) OWM (F-O)	8,62	6,65	3,50	Bif (1), Cl (8)	*B. animalis*, NRI^{S}
PR37	Putty-nosed Monkey (*Cercopithecus nictitans*) OWM (F-O)	8,46	5,46	3,57	Bif (1), Cl (13)	*B. animalis*, NRI^{S}
PR38	Northern Talapoin Monkey (*Miopithecus oguensis*) OWM (F-O)	9,08	6,70	5,39	Cl (9)	NRI^{S}
PR39	De Brazza´s Monkey (*Cercopithecus neglectus*) OWM (F-O)	9,78	7,40	4,86	Bif (1), Cl (8)	*B. angulatum/merycicum*, NRI^{S}
PR40	Northern white-cheeked Gibbon (*Nomascus leucogenys*) OWM (F-F)	9,08	6,60	6,16	Cl (6)	NRI^{S}
PR41	Chimpanzee (*Pan troglodytes*) OWM (F-O)	8,14	8,11	2,00	Bif (4)	*B. dentium*
PR42	Chimpanzee (*Pan troglodytes*) OWM (F-O)	8,60	8,34	2,60	Bif (3)	*B. dentium*
PR43	Chimpanzee (*Pan troglodytes*) OWM (F-O)	8,70	8,41	2,30	Bif (1)	*B. cate/pseudocatenulatum*
PR44	Chimpanzee (*Pan troglodytes*) OWM (F-O)	8,45	8,44	2,60	Bif (3)	*B. dentium*
PR45	Patas Monkey (*Erythrocebus patas*) OWM (F-O)	7,54	7,13	7,28	Bif (4), Cl (2)	*B. angulatum/merycicum*, NRI, NRI^{S}

PR46	Southern Yellow-cheeked Gibbon (*Nomascus gabriellae*) [OWM (F-F)]	8,51	7,66	4,39	Cl (7)	NRI^{S}
PR47	Southern Yellow-cheeked Gibbon (*Nomascus gabriellae*) [OWM (F-F)]	8,10	7,37	5,08	Bif (1), Cl (4)	NRI, NRI^{S}
PR51	Southern Yellow-cheeked Gibbon (*Nomascus gabriellae*) [OWM (F-F)]	8,40	5,30	4,51	Bif (2), Cl (6)	*B. globosum*, NRI^{S}
PR52	Green Monkey (*Chlorocebus sabaeus*) [OWM (F-O)]	8,74	6,45	4,15	Bif (1), Cl (2)	*B. globosum*, NRI^{S}
PR55	Hamlyn’s Monkey (*Cercopithecus hamlyni*) [OWM (F-O)]	7,10	6,87	2,00	Cl (8)	NRI^{S}
PR56	Roloway Monkey (*Cercopithecus roloway*) [OWM (F-O)]	9,02	7,08	2,00	Cl (5)	NRI^{S}
PR57	Lesser spot-nosed Monkey (*Cercopithecus petaurista*) [OWM (F-O)]	7,00	7,00	2,00	Cl (6)	NRI^{S}
PR58	Southern Yellow-cheeked Gibbon (*Nomascus gabriellae*) [OWM (F-F)]	9,30	6,75	2,00	Bif (2)	*B. adolescentis*
PR59	Northern white-cheeked Gibbon (*Nomascus leucogenys*) [OWM (F-F)]	8,58	6,76	2,00	Cl (6)	NRI^{S}
PR60	Northern white-cheeked Gibbon (*Nomascus leucogenys*) [OWM (F-F)]	8,35	4,75	2,78	Cl (9)	NRI^{S}
PR61	Golden Lion Tamarin (*Leontopithecus rosalia*) [NWM (F-I)]	9,26	9,46	9,04	Bif (4)	*B. adolescentis*, *B. stellenboschense*

List of primate hosts (n = 52) with cultivation bacterial counts (log CFU g^{-1}) after microbiological analysis on three different media and subsequent colony isolation. Based on the morphological variability of colonies, the isolates were collected from selective media and then identified to genus/species level (phosphoketolase test, MALDI-TOF mass spectrometry). NWM, New World monkey; OWM, Old World monkey; G-I, gummivore-insectivore; F-O, frugivore-omnivore; F-I, frugivore-insectivore; F-F, frugivore-folivore; WSP, medium for total counts of anaerobic bacteria; MUP, selective medium with mupirocin and acetic acid; NORF, selective medium with norfloxacin, mupirocin, and acetic acid; Bif, bifidobacteria; Cl, clostridia; NRI, not reliable identification; NRI^{S}, not reliable identification with sarcina morphology

Supplementary Table 2: Species MALDI-TOF MS assignment verification by 16S rRNA sequencing

Code	Isolate	Faecal sample of	MALDI-TOF MS identification	16s rRNA gene identification (EZ Taxon)	Identification relation
N2	11/8 NB	Silvery Marmoset	*B. goeldii* (2.08)	*B. goeldii* (99.58 %)	Compliance
N5	18/8A	Chimpanzee	*B. dentium* (2.30)	*B. dentium* (98.74 %)	Compliance
N7	20/6NA	Golden-bellied Mangabey	*B. angulatum/merycicum* (1.81)	*B. angulatum* (99.72 %)	Compliance
N12	22/6E	Lion-tailed Macaque	*B. dentium* (2.23)	*B. dentium* (100.00 %)	Compliance
N13	23/4NC	Hamadryas Baboon	*B. catenulatum/pseudocate.* (2.15)	*B. pseudocatenulatum* (99.51 %)	Compliance
N14	1/8Ba	Common Marmoset	*B. myosotis* (1.95)	*B. myosotis* (99.49 %)	Compliance
N15	15/7ND	Silvery Marmoset	*B. imperatoris/saguini* (2.21)	*B. saguini* (98.94 %)	Compliance
N16	15/7G	Silvery Marmoset	*B. parmae* (2.20)	SEQ_F	-
N22	41/7E	Chimpanzee	*B. dentium* (2.29)	*B. dentium* (98.88 %)	Compliance
N23	42/7C	Chimpanzee	*B. dentium* (2.31)	*B. dentium* (98.86 %)	Compliance
N25	44/8B	Chimpanzee	*B. dentium* (2.44)	*B. dentium* (98.88 %)	Compliance
N30	37/2NB	Putty-nosed Monkey	*B. animalis* (2.49)	*B. animalis* subsp. *lactis* (100.00 %)	Compliance
N33	30/8NC	Goeldi's Marmoset	NRI (1.65)	PGLQ_s (100.00 %)	Compliance
N35	31/8DX	White-headed Marmoset	*B. callitrichos* (2.03)	*B. callitrichos* (99.08 %)	Compliance
N36	32/8B	White-headed Marmoset	*B. callitrichos* (2.04)	*B. callitrichos* (99.57 %)	Compliance
N37	32/8NB	White-headed Marmoset	*B. callitrichos* (1.95)	*B. callitrichos* (98.86 %)	Compliance
N40	33/8NC	Moustached Tamarin	NRI (1.56)	*B. imperatoris* (98.22 %)	Difference
N42	35/9ND	Silvery Marmoset	*B. ramosum* (2.14)	*B. ramosum* (99.78 %)	Compliance

N43	35/9C b	Silvery Marmoset	*B. tissieri* (2.40)	*B. tissieri* (99.65 %)	Compliance
N45	36/2NE	Campbell's Mona Monkey	*B. animalis* (2.46)	*B. animalis* subsp. *lactis* (100.00 %)	Compliance
**N50**	**6/8B**	**Brown-mantled Tamarin**	***B. imperatoris/saguini* (1.87)**	**PEBJ_s (97.52 %)**	Difference/**Pot. nov. sp.**
N55	24/8X	Pygmy Marmoset	*B. aesculapii* (2.13)	*B. aesculapii* (98.45 %)	Compliance
N58	28/9A	Common Marmoset	*B. callitrichos* (1.85)	*B. callitrichos* (99.15 %)	Compliance
N61	45/6B	Patas Monkey	*B. angulatum/merycicum* (1.96)	*B. angulatum* (99.37 %)	Compliance
N63	61A/8B	Golden Lion Tamarin	*B. adolescentis* (2.23)	*B. adolescentis* (99.44 %)	Compliance
N70	28/8NB	Common Marmoset	*B. hapali* (2.41)	SEQ_F	-
N71	28/7D	Common Marmoset	*B. myosotis* (2.12)	*B. myosotis* (99.48 %)	Compliance
N72	51/3NA	Southern Yellow-cheeked Gibbon	*B. globosum* (2.37)	*B. pseudolongum* subsp. *globosum* (99.93 %)	Compliance
**N74**	**4/7 NC**	**Emperor Tamarin**	**NRI (1.41)**	***B. myosotis* (96.41 %)**	Compliance/**Pot. nov. sp.**
N81	33/1	Moustached Tamarin	*B. parmae* (2.26)	SEQ_F	-
N83	33/7	Moustached Tamarin	*B. imperatoris/saguini* (2.16)	*B. saguini* (99.58 %)	Compliance
N91	11/7NC	Silvery Marmoset	*B. imperatoris/saguini* (2.14)	*B. saguini* (99.01 %)	Compliance
**N94**	**26/6NB**	**Cotton-top Tamarin**	**NRI (1.56)**	***B. avesanii* (95.68 %)**	Compliance/**Pot. nov. sp.**
**N97**	**33/9NA**	**Moustached Tamarin**	**NRI (1.52)**	***B. aerophilum* (97.38 %)**	Compliance/**Pot. nov. sp.**
N98	34/7NC	Patas Monkey	*B. angulatum/merycicum* (1.91)	*B. angulatum* (99.36 %)	Compliance
N99	35/9NB	Silvery Marmoset	*B. callitrichos* (2.16)	*B. callitrichos* (99.50 %)	Compliance
N100	52/2NA	Green Monkey	*B. globosum* (2.36)	*B. pseudolongum* subsp. *globosum* (99.58 %)	Compliance
N106	24/7D	Pygmy Marmoset	*B. ramosum* (2.17)	*B. ramosum* (99.13 %)	Compliance
N113	5/8F	Moustached Tamarin	*B. vansiderenii* (2.26)	*B. vansinderenii* (100.00 %)	Compliance
**N115**	**7/8NA**	**Red-handed Tamarin**	***B. stellenboschense* (2.10)**	***B. stellenboschense* (96.28%)**	Compliance/**Pot. nov. sp.**
N119	24/9B	Pygmy Marmoset	*B. reuteri* (2.17)	SEQ_F	-
N121	10/8B	Silvery Marmoset	*B. imperatoris/saguini* (2.16)	*B. saguini* (99.57 %)	Compliance
N123	19/6 E-2	Northern White-cheeked Gibbon	*B. catenulatum/pseudocate.* (2.32)	*B. pseudocatenulatum* (99.29 %)	Compliance
N124	26/7A	Cotton-top Tamarin	*B. callitrichidarum* (2.14)	*B. callitrichidarum* (98.91 %)	Compliance
N125	27/8NE	Golden Lion Tamarin	*B. parmae* (2.11)	SEQ_F	-
N127	35/9NA	Silvery Marmoset	*B. adolescentis* (2.28)	*B. faecale* (99.23 %)	**Difference**

Verification of MALDI-TOF MS bifidobacterial identification by 16S rRNA gene sequencing. NRI, not reliable identification; Pot. nov. sp., Potential novel species of bifidobacteria; SEQ_F, sequencing failed; *B. catenulatum/pseudocate.*, *B.catenulatum/pseudocatenulatum.*

Supplementary Table 3. List of BRUKER and new database entries (MSPs) for bifidobacterial MALDI-TOF MS identification

	No.	Species/subspecies	Strain code	Origin
New database entries (MSPs)	1	*Bifidobacterium actinocolonuiiforme*	DSMZ 22766	Bumblebee (*Bombus lucorum*) digestive tract
	2	*Bifidobacterium aemilianum*	LMG 30143	Carpenter bee (*Xylocopa violacea*)
	3	*Bifidobacterium aerophilum*	DSM 100689	Faeces of an adult cotton-top tamarin *Saguinus oedipus* L.
	4	*Bifidobacterium aesculapii*	DSMZ 26737	Marmosets faeces (*Callithrix jacchus*)
	5	*Bifidobacterium anseris*	LMG 30189	*Anser domesticus*
	6	*Bifidobacterium apri*	DSMZ 100238	Large intestine contents of a wild boar (*Sus scrofa scrofa*)
	7	*Bifidobacterium aquikefiri*	LMG 28769	Water kefir
	8	*Bifidobacterium avesanii*	DSMZ 100685	Faeces of an adult cotton-top tamarin *Saguinus oedipus* L.
	9	*Bifidobacterium biavatii*	DSMZ 23969	Faeces of tamarin (red-handed marmoset)
	10	*Bifidobacterium bohemicum*	DSMZ 22767	Bumblebee (*Bombus lucorum*) digestive tract
	11	*Bifidobacterium bombi*	DSMZ 19703	Digestive tract content of the bumblebee *Bombus lucorum*
	12	*Bifidobacterium callimiconis*	LMG 30938	Goeldi's monkey (*Callimico goeldii*)
	13	*Bifidobacterium callitrichidarum*	DSMZ 103152	Faeces of *Saguinus imperator*
	14	*Bifidobacterium callitrichos*	DSMZ 23973	Faeces of common marmoset
	15	*Bifidobacterium castoris*	LMG 30937	Beaver (*Castor fiber*)
	16	*Bifidobacterium catenulatum* subsp. *kashiwanohense*	DSMZ 21854	Faeces of a healthy infant (male; 1.5 years old)
	17	*Bifidobacterium catulorum*	DSMZ 103154	Faeces of *Callithrix jacchus*
	18	*Bifidobacterium criceti*	LMG 30188	*Cricetus cricetus*
	19	*Bifidobacterium crudilactis*	LMG 23609	Raw cow milk
	20	*Bifidobacterium cuniculi*	DSMZ 20435	Rabbit faeces
	21	*Bifidobacterium dolichotidis*	LMG 30941	Patagonian Cavy (*Dolichotis patagonum*)
	22	*Bifidobacterium eulemuris*	DSMZ 100216	Fresh faeces of black lemurs *Eulemur macaco* housed under semi-natural conditions
	23	*Bifidobacterium faecale*	LMG 30642	Faeces of two-week-old baby
	24	*Bifidobacterium globosum*	DSMZ 20092	Rumen
	25	*Bifidobacterium goeldii*	LMG 30939	Goeldi's monkey (*Callimico goeldii*)
	26	*Bifidobacterium hapali*	DSMZ 100202	Faeces of baby common marmosets (*Callithrix jacchus* L.)
	27	*Bifidobacterium imperatoris*	LMG 30297	Emperor tamarin
	28	*Bifidobacterium indicum*	DSMZ 20214	Hindgut of honeybee
	29	*Bifidobacterium italicum*	LMG 30187	Emperor tamarin, faeces
	30	*Bifidobacterium lemurum*	DSMZ 28807	Faeces of a 5-year-old ring-tailed lemur (*Lemur catta*)
	31	*Bifidobacterium longum* subsp. *suillum*	DSMZ 28597	Faeces of piglets
	32	*Bifidobacterium margollesii*	LMG 30296	*Callithrix pygmaea*
	33	*Bifidobacterium mongoliense*	DSMZ 21395	Airag, the Mongolian traditional beverage made of fermented mare's milk
	34	*Bifidobacterium moukalabense*	DSMZ 27321	Faeces of wild western lowland gorilla (*Gorilla gorilla gorilla*)
	35	*Bifidobacterium myosotis*	DSMZ 100196	Faeces of baby common marmosets (*Callithrix jacchus* L.)
	36	*Bifidobacterium parmae*	LMG 30295	*Callithrix pygmaea*
	37	*Bifidobacterium porcinum*	DSMZ 17755	Faeces of piglet
	38	*Bifidobacterium pseudolongum*	DSMZ 20099	Pig faeces
	39	*Bifidobacterium psychraerophilum*	DSMZ 22366	Pig caecum
	40	*Bifidobacterium ramosum*	DSMZ 100688	Faeces of an adult cotton-top tamarin (*Saguinus oedipus* L.)
	41	*Bifidobacterium reuteri*	DSMZ 23975	Faeces of common marmoset

	42	*Bifidobacterium saguini*	DSMZ 23967	Faeces of tamarin (red-handed marmoset)
	43	*Bifidobacterium samirii*	LMG 30940	Black-capped squirrel monkey (*Saimiri boliviensis peruviensis*)
	44	*Bifidobacterium stellenboschense*	DSMZ 23968	Faeces of tamarin (red-handed marmoset)
	45	*Bifidobacterium subtile*	DSMZ 20096	Sewage
	46	*Bifidobacterium thermacidophilum*	DSMZ 15837	Waste water of a bean-curd farm
	47	*Bifidobacterium tissieri*	DSMZ 100201	Faeces of baby common marmosets (*Callithrix jacchus* L.)
	48	*Bifidobacterium tsurumiense*	DSMZ 17777	Hamster, dental plaque
	49	*Bifidobacterium vansinderenii*	LMG 30126	Emperor tamarin (*Saguinus imperator*)
	50	*Bifidobacterium xylocopae*	LMG 30142	Carpenter bee (*Xylocopa violacea*)
**BRUKER entries (MSPs)**	1	*Bifidobacterium adolescentis*		
	2	*Bifidobacterium angulatum*		
	3	*Bifidobacterium animalis*		
	4	*Bifidobacterium asteroides*		
	5	*Bifidobacterium bifidum*		
	6	*Bifidobacterium boum*		
	7	*Bifidobacterium breve*		
	8	*Bifidobacterium catenulatum*		
	9	*Bifidobacterium choerinum*		
	10	*Bifidobacterium coryneforme*		
	11	*Bifidobacterium dentium*		
	12	*Bifidobacterium gallicum*		
	13	*Bifidobacterium gallinarum*		
	14	*Bifidobacterium longum*		
	15	*Bifidobacterium magnum*		
	16	*Bifidobacterium merycicum*		
	17	*Bifidobacterium minimum*		
	18	*Bifidobacterium pseudocatenulatum*		
	19	*Bifidobacterium pseudolongum*		
	20	*Bifidobacterium pullorum*		
	21	*Bifidobacterium ruminantium*		
	22	*Bifidobacterium saeculare*		
	23	*Bifidobacterium scardovii*		
	24	*Bifidobacterium thermoacidophilum*		
	25	*Bifidobacterium thermophilum*		

By mid-2019 MALDI-TOF MS database contained 25 bifidobacterial species (Bruker Daltonik GmbH, Bremen, Germany). For rapid identification of wild bifidobacterial strains, this database was expanded by currently available 50 species of bifidobacteria. All new entries were type strains obtained from Deutsche Sammlung von Mikroorganismen und Zellkulturen (DSMZ, Germany) or Belgian Coordinated Collections of Microorganisms (BCCM/LMG, Belgium). Main spectra profiles (MSPs) were prepared from freshly grown culture on non-selective Reinforced Clostridial Agar (Merck, Darmstadt, Germany) under anaerobic conditions (80 % N_2, 10 % CO_2, 10 % H_2; Don Whitley Scientific, Bingley, UK) at 37 °C for 2 days. These samples were prepared with ethanol-formic acid extraction procedure with HCCA matrix solution (α-cyano-4-hydroxycinnamic acid; Bruker) according to the manufacturer's instructions. Cells were harvested and transferred to 300 µL of deionized water followed by addition of 900 µL of absolute ethanol (VWR) and thorough mixing. After centrifugation (14 500 rpm per 2 min) and supernatant removal, pellets were allowed to dry. Thereafter, the pellets were resuspended in 70% formic acid (Carl Roth, Karlsruhe, Germany) and the same concentration of acetonitrile (VWR) and centrifuged for 2 min at 14 500 rpm. The resulting supernatant was applied onto a target and overlaid with HCCA matrix. To ensure a high spectrum quality and reproducibility, twenty-four replicates for each entry were obtained. Subsequently, a quality check was performed with FlexAnalysis software, and the spectra were uploaded with MSP generation into the in-home database with MBT Compass Explorer (both Bruker). DSMZ, Deutsche Sammlung von Mikroorganismen und Zellkulturen; LMG, Belgian Coordinated Collections of Microorganisms.

Supplementary S1

March 25, 2021

1 Diversity statistics

[1]:
```
import sys
import copy

sys.path.append("/home/user/Programs/seqDataClassCluster")

import pandas as pd
import numpy as np
import random

import plotly.express as px
import plotly.graph_objs as go
from plotly.subplots import make_subplots

from seqDataClass import seqObject
#import seqDataClass

template = 'plotly_white'

filepath = "/home/user/qiime/monkeys/"

seq1 = seqObject(mappingFile = filepath+"mapping file_monkeys_simplified.csv",
                 taxonomyFile = filepath+"dada2_taxonomy_updated.csv",
                 featureFile = filepath+"dada2_feature_table_nochim.csv",
                 mappingSep = ";",
                 taxonomySep = ",",
                 featureSep = ",",
                 sampleNamesColumn = "sample-id",
                 #featureFormat = "dada2",
                 #taxonomyFormat = "dada2"
                 )

seq1.extract_rep_seq(filename=filepath+"rep_seq.fasta", renameFeatures=True)

seq1.rarefy_to_even_depth(seqDepth=42134, seed=123)
```

```python
seq1.extract_features(tax_level="feature-id")
```

```
Initializing data loading.
Loading mapping file
Loading taxonomy
Recognizing a DADA2 format.
Matching features between feature file and taxonomy file.
Features match.
Removing 2513 features/OTUs that no longer appear in any sample.
```

[1]:
```
sample-id      PR1  PR10  PR11   PR15  PR16  PR17  PR18  PR19   PR2  PR20  \
feature-id
feature0      1577  3831  3820   1722  3188  3392  1732  1062  1699   670
feature1       472  5284  5531   1035  2800  4237  1193   936   715   591
feature2        97  2080  1865   1142  1361  2715  1127   204   327   360
feature3      4770    83    59    186   441   232  1064  1023  2554  1222
feature4       288   803  1425  14955  5788  2055   637   110    94    12
…              …    …    …    …    …    …    …    …    …    …
feature11941     0     0     0      0     0     0     0     0     0     0
feature11942     0     0     0      0     0     0     0     0     0     0
feature11946     0     0     0      0     0     0     0     0     0     0
feature11947     0     0     0      0     0     0     0     0     0     0
feature11949     0     0     0      0     0     0     0     0     0     0

sample-id     …  PR56  PR57  PR58  PR59   PR6  PR60  PR61   PR7   PR8   PR9
feature-id    …
feature0      …   116    60   161   103  2543    66   628  3469  1993  4764
feature1      …   220    17    89   133  2220    10  2211  2626  1277  4694
feature2      …   183    10   180   411  4181     8   187  4909  2857  1866
feature3      …   145   359  1048   523   194  1629  2001    91   282   160
feature4      …    45   671   199    58   437     2   138   687   873  1300
…            …   …    …    …    …    …    …    …    …    …    …
feature11941  …     0     0     0     0     0     0     0     0     0     1
feature11942  …     0     0     0     0     0     0     0     0     0     2
feature11946  …     0     0     0     0     0     0     0     0     0     1
feature11947  …     0     0     0     0     0     0     0     0     0     3
feature11949  …     0     0     0     0     0     0     0     0     0     2

[9439 rows x 52 columns]
```

Following is a diversity analysis between the locations. I have focused on Feature richness, which is direct ASV count (formerly OTU count), Shannon diversity/entropy and Pielou's evennes.

[2]:
```python
from skbio.diversity import alpha

import statsmodels.api as sm
from statsmodels.formula.api import ols, wls, rlm
```

```
import scipy as sp
```

```
/home/user/Programs/anaconda3/envs/bioconda/lib/python3.6/site-
packages/skbio/util/_testing.py:16: FutureWarning:

pandas.util.testing is deprecated. Use the functions in the public API at
pandas.testing instead.
```

[3]:
```
df1 = seq1.extract_features(map_level="sample-id", tax_level="feature-id")

diversity = []
for sample in df1:
    diversity.append({"index": sample, "feature_count":alpha.
 ↪observed_otus(df1[sample]), "shannon":alpha.shannon(df1[sample]), "pielou":␣
 ↪alpha.pielou_e(df1[sample])})

df2 = pd.DataFrame(diversity)
df2.index = df2["index"]
del df2["index"]

df3 = seq1.data.columns.to_frame()
df3.index = df3["sample-id"]

df2 =  pd.concat([df3, df2], axis=1)

new_columns = list(df2.columns)
new_columns[3] = "sub_order"
df2.columns = new_columns
```

1.1 Diversity per sub-order of primates

[4]:
```
fig = make_subplots(rows = 1, cols = 3, shared_yaxes=False, subplot_titles =␣
 ↪["Feature count", "Shannon diversity", "Pielou evenness"])

x = df2["sub_order"]

#df3 = df2[df2["Location"] == "Arid"]
fig.add_trace(go.Box(y=df2["feature_count"], x=x, showlegend=False), row = 1,␣
 ↪col = 1)
fig.add_trace(go.Box(y=df2["shannon"], x=x, showlegend=False), row = 1, col = 2)
fig.add_trace(go.Box(y=df2["pielou"], x=x, showlegend=False), row = 1, col = 3)
#df3 = df2[df2["Location"] == "Semi-arid"]
#fig.add_trace(go.Box(y=df4["shannon"], x=x, name="Zygophylum"))
```

```
fig.update_layout(template=template,
                  title="Diversity per sub-order",
                  font_color="black",
                  width=800,
                  height=600
                  )
fig.show()
fig.write_image("final_files/diversity_suborder.pdf",height = 400, width = 700)
```

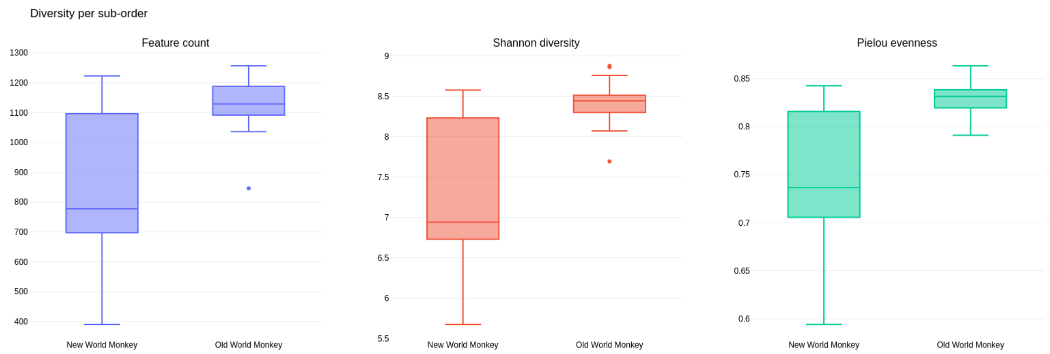

1.1.1 Difference in variance adjustment

Heteroskedasticity adjustment

[5]:
```
df_var = df2.groupby(["sub_order"]).var()

new_row = {'feature_count': df_var.loc["New World Monkey", "feature_count"]/
 ↪df_var.loc["Old World Monkey", "feature_count"],
           'shannon':df_var.loc["New World Monkey", "shannon"]/df_var.loc["Old␣
 ↪World Monkey", "shannon"],
           'pielou':df_var.loc["New World Monkey", "pielou"]/df_var.loc["Old␣
 ↪World Monkey", "pielou"]}
df_var = df_var.append(new_row, ignore_index=True)
df_var.index = ["New World Monkey Variance", "Old World Monkey Variance",␣
 ↪"Between group proportion"]
df_var
```

[5]:

	feature_count	shannon	pielou
New World Monkey Variance	49914.862319	0.763738	0.004306
Old World Monkey Variance	6817.698413	0.055951	0.000267
Between group proportion	7.321366	13.650181	16.128088

The varance between the tested groups is between 7 to 16 times larger for one group than the

other. This severely violates the assumption of ordinary statistical methods. Wilcoxon's test is also not applicable since the groups have different number of memebers. To resolve this issue, all three datasets (feature count, Shannon diversity and Pielou evenness) will be Box-Cox transfromed before the analysis. Furthermore, the linear models are constructed using MacKinnon and White's (1985) heteroskedasticity robust standard errors.

1.1.2 Feature count statistics

In the following section, we are testing whether there is a statistically significant difference between the means of the new world monkeys and old worls monkeys gut microbiota. The null hypothesis is that there is no difference between the two grup means.

[6]:
```
fig = go.Figure()

x = df2["sub_order"]

fig.add_trace(go.Box(y=df2["feature_count"], x=x, showlegend=False))
#fig.add_trace(go.Box(y=df2["shannon"], x=x, showlegend=False), row = 1, col =␣
↪2)
#fig.add_trace(go.Box(y=df2["pielou"], x=x, showlegend=False), row = 1, col = 3)
fig.update_layout(template=template, title="Feature count per sub-order",␣
↪width=400, height=600)

fig.show()
```

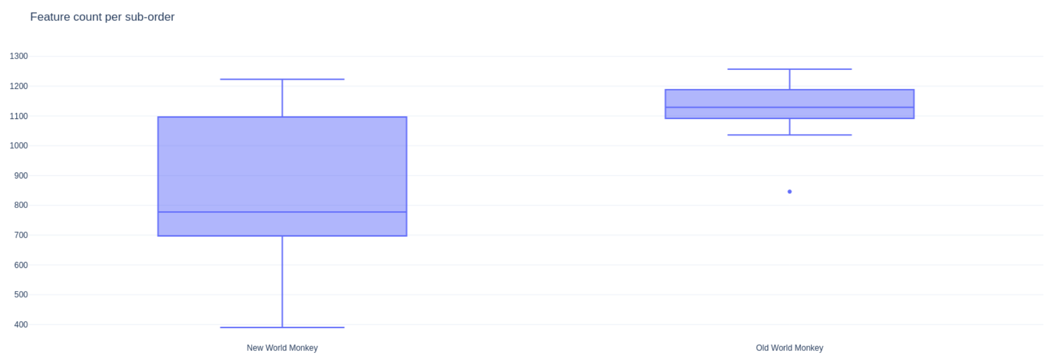

[7]:
```
df2["feature_count_boxcox"], bc_lambda = sp.stats.boxcox(df2["feature_count"])
print(f"Box-Cox lambda: {bc_lambda:.3f}")
```

```
Box-Cox lambda: 3.295
```

[8]:
```
mod = ols("feature_count_boxcox ~ sub_order", data=df2).fit(cov_type='HC1')
print(mod.summary())
```

```
                            OLS Regression Results
================================================================================
Dep. Variable:     feature_count_boxcox   R-squared:                       0.398
Model:                              OLS   Adj. R-squared:                  0.385
Method:                   Least Squares   F-statistic:                     30.47
Date:                  Thu, 25 Mar 2021   Prob (F-statistic):           1.21e-06
Time:                          11:18:23   Log-Likelihood:                -1155.1
No. Observations:                    52   AIC:                             2314.
Df Residuals:                        50   BIC:                             2318.
Df Model:                             1
Covariance Type:                    HC1
================================================================================
=================
                                   coef    std err          z      P>|z|
[0.025      0.975]
--------------------------------------------------------------------------------
-----------------
Intercept                     1.796e+09   2.81e+08      6.395      0.000
1.25e+09    2.35e+09
sub_order[T.Old World Monkey]  1.749e+09   3.17e+08      5.520      0.000
1.13e+09    2.37e+09
==============================================================================
Omnibus:                        2.661   Durbin-Watson:                   1.208
Prob(Omnibus):                  0.264   Jarque-Bera (JB):                2.553
Skew:                           0.495   Prob(JB):                        0.279
Kurtosis:                       2.555   Cond. No.                         2.72
==============================================================================

Warnings:
[1] Standard Errors are heteroscedasticity robust (HC1)
```

- Distribution : The Jarqu-Bera test probability is 0.279, which means that we cannot reject the null hypothesis that the residuals are normally distributed. This is also supported by the results of the Omnibus test (probability 0.264) as well as a visual inspection of the residuals in a qq-plot above.

[9]:
```
aov_table = sm.stats.anova_lm(mod, typ=2)
eta_sqrd = aov_table['sum_sq'][0]/
↪(aov_table['sum_sq'][0]+aov_table['sum_sq'][1])

print(f"F({aov_table['df'][0]:.0f}, {aov_table['df'][1]:.0f}) =␣
↪{aov_table['F'][0]:.2f}, p = {aov_table['PR(>F)'][0]:.2e}, \N{GREEK SMALL␣
↪LETTER ETA}\u00b2 = {eta_sqrd:.3}")
```

```
F(1, 50) = 30.47, p = 1.21e-06,  ² = 0.379
```

Test statistic result follow bellow. The result is significant, which means that **we can reject the null hypothesis** that the two groups have the same population mean. In other words the feature counts are statistically significantly different in the two groups.

[10]:
```python
fig = sm.qqplot(mod.resid, fit=True, line="45")
```

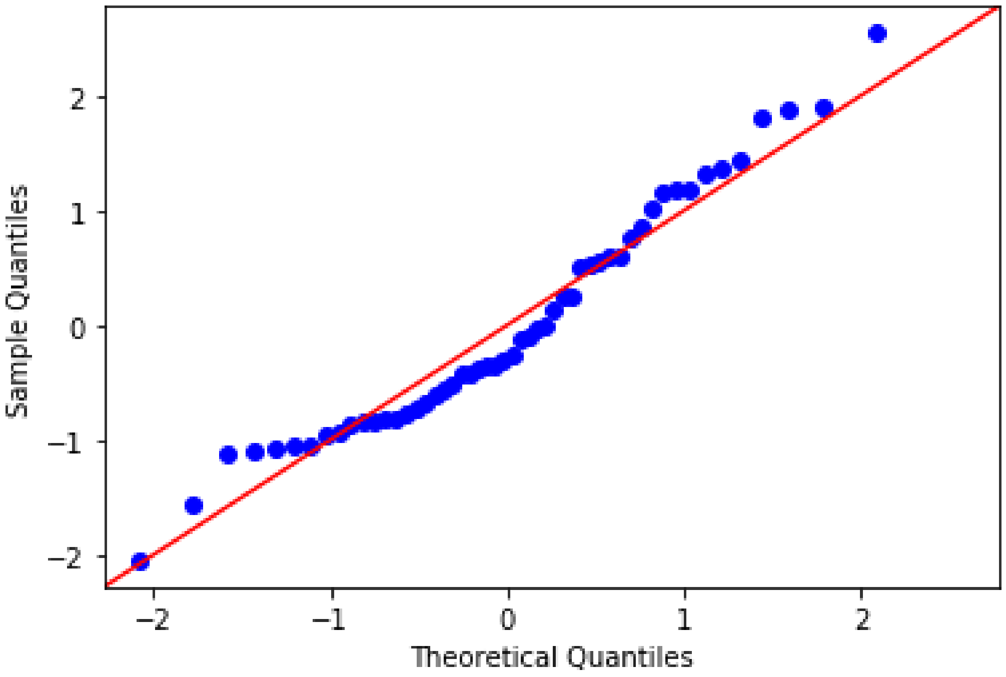

The test result is statistically significant, which means that we can reject the null hypothesis that the residuals are homoskedastic and confirm the previous observation. In order to account for that, we will use White-Huber-Eicker standard errors since we do not know the form of heteroskedasticity. This method is specifically robust against heteroschedasticity of the residuals.

There is a statistically significant difference in the ALS counts between the primate sub-orders $F(1{,}50) = 30.31$, $p = 1.27{\times}10\text{-}6$, $^{2} = 0.38$.

With this adjustment, the model assumptions have been met.

1.1.3 Shannon diversity statistics

[11]:
```python
fig = go.Figure()

x = df2["sub_order"]

fig.add_trace(go.Box(y=df2["shannon"], x=x, showlegend=False))
#fig.add_trace(go.Box(y=df2["shannon"], x=x, showlegend=False), row = 1, col =␣
↪2)
#fig.add_trace(go.Box(y=df2["pielou"], x=x, showlegend=False), row = 1, col = 3)
fig.update_layout(template=template, title="Shannon diversity per sub-order",␣
↪width=400, height=600)
```

```
fig.show()
```

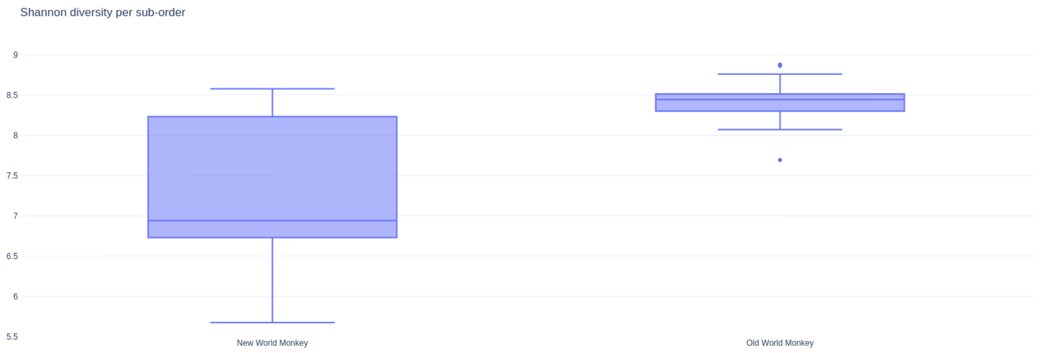

[12]:
```
df2["shannon_boxcox"], bc_lambda = sp.stats.boxcox(df2["shannon"])
print(f"Box-Cox lambda: {bc_lambda:.3f}")
```

```
Box-Cox lambda: 7.698
```

[13]:
```
mod = ols('shannon_boxcox ~ sub_order', data=df2).fit(cov_type='HC1')
print(mod.summary())
```

```
                            OLS Regression Results
==============================================================================
Dep. Variable:         shannon_boxcox   R-squared:                       0.452
Model:                            OLS   Adj. R-squared:                  0.441
Method:                 Least Squares   F-statistic:                     38.01
Date:                Thu, 25 Mar 2021   Prob (F-statistic):           1.21e-07
Time:                        11:18:23   Log-Likelihood:                -757.41
No. Observations:                  52   AIC:                             1519.
Df Residuals:                      50   BIC:                             1523.
Df Model:                           1
Covariance Type:                  HC1
================================================================================
=================
                                    coef    std err          z      P>|z|
[0.025      0.975]
--------------------------------------------------------------------------------
-----------------
Intercept                      8.185e+05   1.35e+05      6.069      0.000
5.54e+05    1.08e+06
sub_order[T.Old World Monkey]  9.335e+05   1.51e+05      6.165      0.000
6.37e+05    1.23e+06
==============================================================================
Omnibus:                        3.882   Durbin-Watson:                   1.342
```

```
Prob(Omnibus):                  0.144   Jarque-Bera (JB):               3.494
Skew:                           0.555   Prob(JB):                       0.174
Kurtosis:                       2.382   Cond. No.                        2.72
==============================================================================

Warnings:
[1] Standard Errors are heteroscedasticity robust (HC1)
```

[14]:
```
fig = sm.qqplot(mod.resid, fit=True, line="45")
```

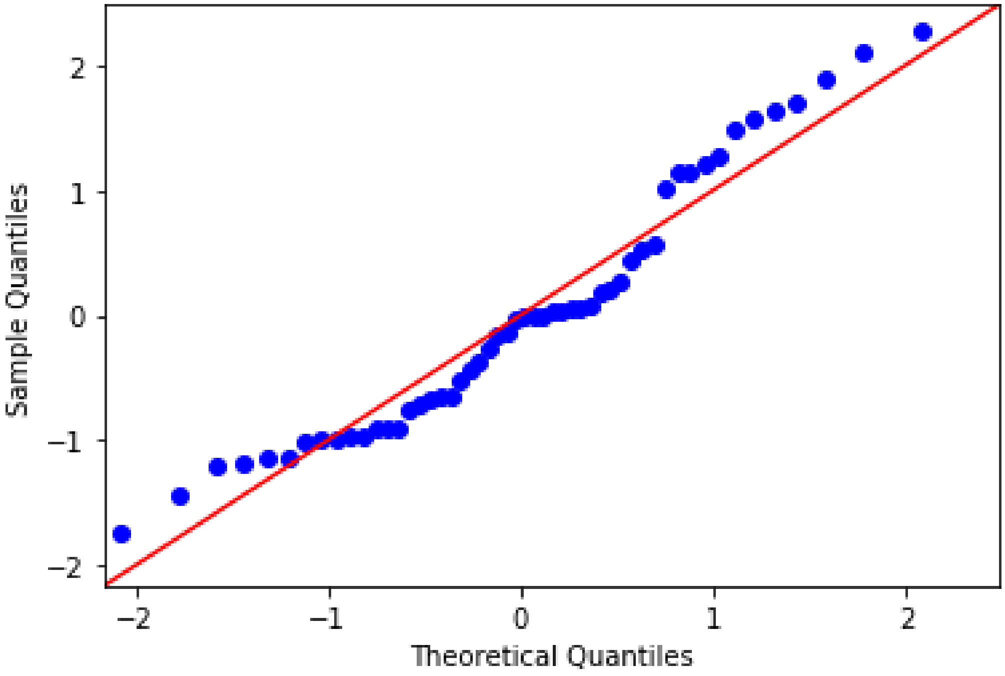

[15]:
```
aov_table = sm.stats.anova_lm(mod, typ=2)
eta_sqrd = aov_table['sum_sq'][0]/
↪(aov_table['sum_sq'][0]+aov_table['sum_sq'][1])

print(f"F({aov_table['df'][0]:.0f}, {aov_table['df'][1]:.0f}) =␣
↪{aov_table['F'][0]:.2f}, p = {aov_table['PR(>F)'][0]:.2e}, \N{GREEK SMALL␣
↪LETTER ETA}\u00b2 = {eta_sqrd:.3}")
```

```
F(1, 50) = 38.01, p = 1.21e-07,  ² = 0.432
```

The test result is statistically significant, which means that we **can reject the null hypothesis that the residuals are homoskedastic**. In order to account for that, we will use White-Huber-Eicker standard errors since we do not know the form of heteroskedasticity. This method is specifically robust against heteroschedasticity of the residuals.

There is a statistically significant difference in the Shannon diversity between the

primate sub-orders

- Distribution : The Jarqu-Bera test probability is 0.174, which means that we cannot reject the null hypothesis that the residuals are not normally distributed. This is also supported by the results of the Omnibus test (probability 0.144) as well as a visual inspection of the residuals in a qq-plot above.

The asssumptions of the models are satisfied with the above mentioned adjustments.

1.1.4 Pielou evenness

[16]:
```
fig = go.Figure()

x = df2["sub_order"]

fig.add_trace(go.Box(y=df2["pielou"], x=x, showlegend=False))
#fig.add_trace(go.Box(y=df2["shannon"], x=x, showlegend=False), row = 1, col =␣
↪2)
#fig.add_trace(go.Box(y=df2["pielou"], x=x, showlegend=False), row = 1, col = 3)
fig.update_layout(template=template, title="Pielou evenness per sub-order",␣
↪width=400, height=600)

fig.show()
```

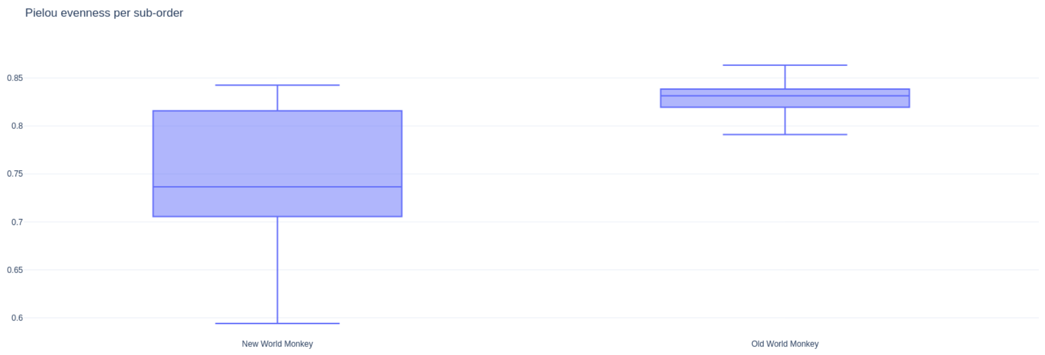

[17]:
```
df2["pielou_boxcox"], bc_lambda = sp.stats.boxcox(df2["pielou"])
print(f"Box-Cox lambda: {bc_lambda:.3f}")
```

```
Box-Cox lambda: 10.853
```

[18]:
```
mod = ols('pielou_boxcox ~ sub_order', data=df2).fit(cov_type='HC1')
print(mod.summary())
```

```
                            OLS Regression Results
```

```
==============================================================================
Dep. Variable:          pielou_boxcox   R-squared:                       0.453
Model:                            OLS   Adj. R-squared:                  0.442
Method:                 Least Squares   F-statistic:                     38.41
Date:                Thu, 25 Mar 2021   Prob (F-statistic):           1.08e-07
Time:                        11:18:24   Log-Likelihood:                 219.26
No. Observations:                  52   AIC:                            -434.5
Df Residuals:                      50   BIC:                            -430.6
Df Model:                           1
Covariance Type:                  HC1
================================================================================
=================
                                   coef    std err          z      P>|z|
[0.025      0.975]
--------------------------------------------------------------------------------
-----------------
Intercept                       -0.0862      0.001    -93.007      0.000
-0.088      -0.084
sub_order[T.Old World Monkey]    0.0065      0.001      6.197      0.000
0.004       0.009
==============================================================================
Omnibus:                        3.500   Durbin-Watson:                   1.447
Prob(Omnibus):                  0.174   Jarque-Bera (JB):                3.069
Skew:                           0.502   Prob(JB):                        0.216
Kurtosis:                       2.362   Cond. No.                         2.72
==============================================================================

Warnings:
[1] Standard Errors are heteroscedasticity robust (HC1)
```

[19]:
```
fig = sm.qqplot(mod.resid, fit=True, line="45")
```

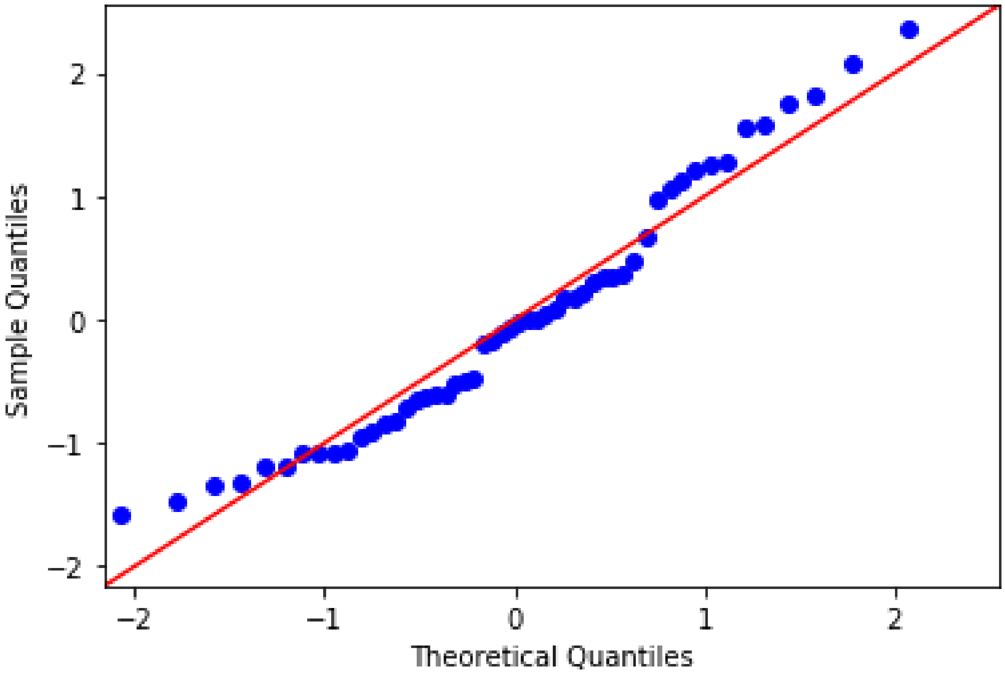

[20]:
```
aov_table = sm.stats.anova_lm(mod, typ=2)
eta_sqrd = aov_table['sum_sq'][0]/
↪(aov_table['sum_sq'][0]+aov_table['sum_sq'][1])

print(f"F({aov_table['df'][0]:.0f}, {aov_table['df'][1]:.0f}) =␣
↪{aov_table['F'][0]:.2f}, p = {aov_table['PR(>F)'][0]:.2e}, \N{GREEK SMALL␣
↪LETTER ETA}\u00b2 = {eta_sqrd:.3}")
```

```
F(1, 50) = 38.41, p = 1.08e-07,  ² = 0.434
```

- Distribution : The Jarqu-Bera test probability is 0.216, which means that we cannot reject the null hypothesis that the residuals are not normally distributed. This is also supported by the results of the Omnibus test (probability 0.174) as well as a visual inspection of the residuals in a qq-plot above.

The asssumptions of the models are satisfied with the above mentioned adjustments.

1.2 Diversity per feed category

[21]:
```
fig = make_subplots(rows = 1, cols = 3, shared_yaxes=False, subplot_titles =␣
↪["Feature count", "Shannon diversity", "Pielou evenness"])

x = df2["feed"]

#df3 = df2[df2["Location"] == "Arid"]
```

```
fig.add_trace(go.Box(y=df2["feature_count"], x=x, showlegend=False), row = 1,␣
 ↪col = 1)
fig.add_trace(go.Box(y=df2["shannon"], x=x, showlegend=False), row = 1, col = 2)
fig.add_trace(go.Box(y=df2["pielou"], x=x, showlegend=False), row = 1, col = 3)
#df3 = df2[df2["Location"] == "Semi-arid"]
#fig.add_trace(go.Box(y=df4["shannon"], x=x, name="Zygophylum"))

fig.update_layout(template=template,
                  title="Diversity per feed category",
                  width=800,
                  height=600,
                  font_color="black",
                 )

fig.show()

fig.write_image("final_files/diversity_feed.pdf",height = 400, width = 700)
```

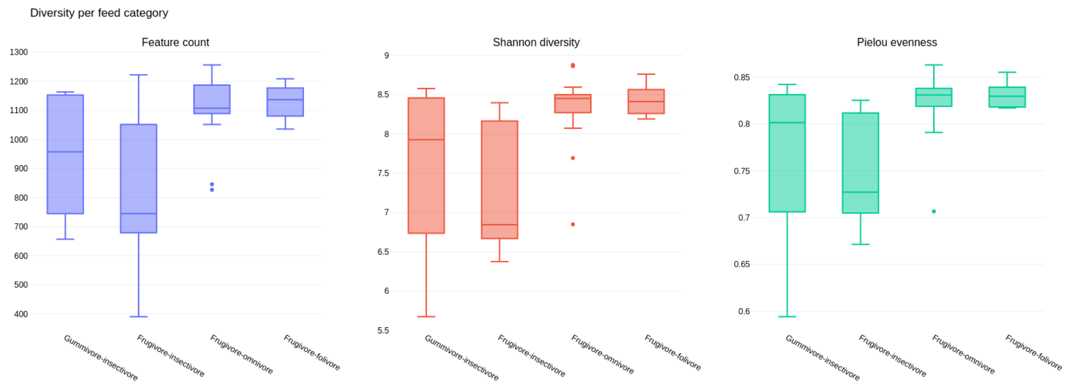

1.2.1 Feature count statistics

[22]:
```
fig = go.Figure()

x = df2["feed"]

fig.add_trace(go.Box(y=df2["feature_count"], x=x, showlegend=False))

fig.update_layout(template=template, title="Feature count per feed category",␣
 ↪width=400, height=600)

fig.show()
```

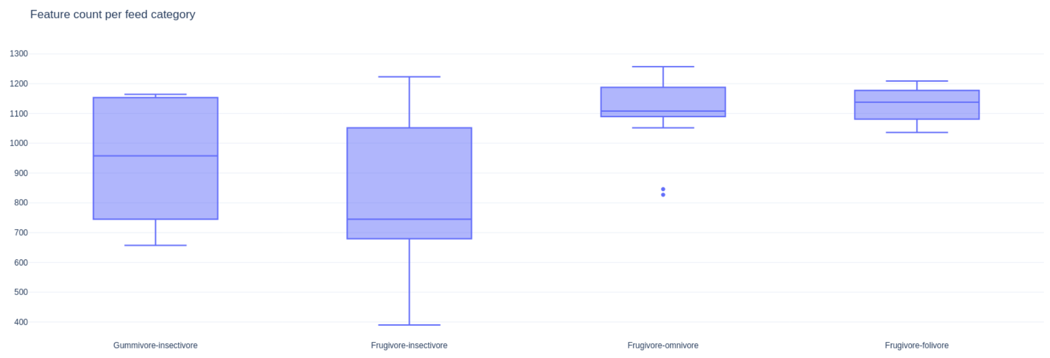

[23]:
```python
mod = ols('feature_count_boxcox ~ feed', data=df2).fit(cov_type='HC1')

print(mod.summary())
```

```
                            OLS Regression Results
==============================================================================
Dep. Variable:     feature_count_boxcox   R-squared:                       0.378
Model:                              OLS   Adj. R-squared:                  0.339
Method:                   Least Squares   F-statistic:                     9.551
Date:                  Thu, 25 Mar 2021   Prob (F-statistic):           4.68e-05
Time:                          11:18:24   Log-Likelihood:                -1155.9
No. Observations:                    52   AIC:                             2320.
Df Residuals:                        48   BIC:                             2328.
Df Model:                             3
Covariance Type:                    HC1
==============================================================================
=================
                                  coef    std err          z      P>|z|
[0.025      0.975]
------------------------------------------------------------------------------
-----------------
Intercept                    3.522e+09   2.19e+08     16.051      0.000
3.09e+09    3.95e+09
feed[T.Frugivore-insectivore] -2.008e+09   4.28e+08     -4.695      0.000
-2.85e+09   -1.17e+09
feed[T.Frugivore-omnivore]    -7.768e+07   3.06e+08     -0.254      0.800
-6.77e+08    5.22e+08
feed[T.Gummivore-insectivore] -1.305e+09   5.09e+08     -2.562      0.010
-2.3e+09   -3.07e+08
==============================================================================
Omnibus:                        0.789   Durbin-Watson:                   1.420
Prob(Omnibus):                  0.674   Jarque-Bera (JB):                0.820
Skew:                           0.272   Prob(JB):                        0.664
```

```
Kurtosis:                       2.713   Cond. No.                         6.10
==============================================================================

Warnings:
[1] Standard Errors are heteroscedasticity robust (HC1)
```

[24]:

```
fig = sm.qqplot(mod.resid, fit=True, line="45")
```

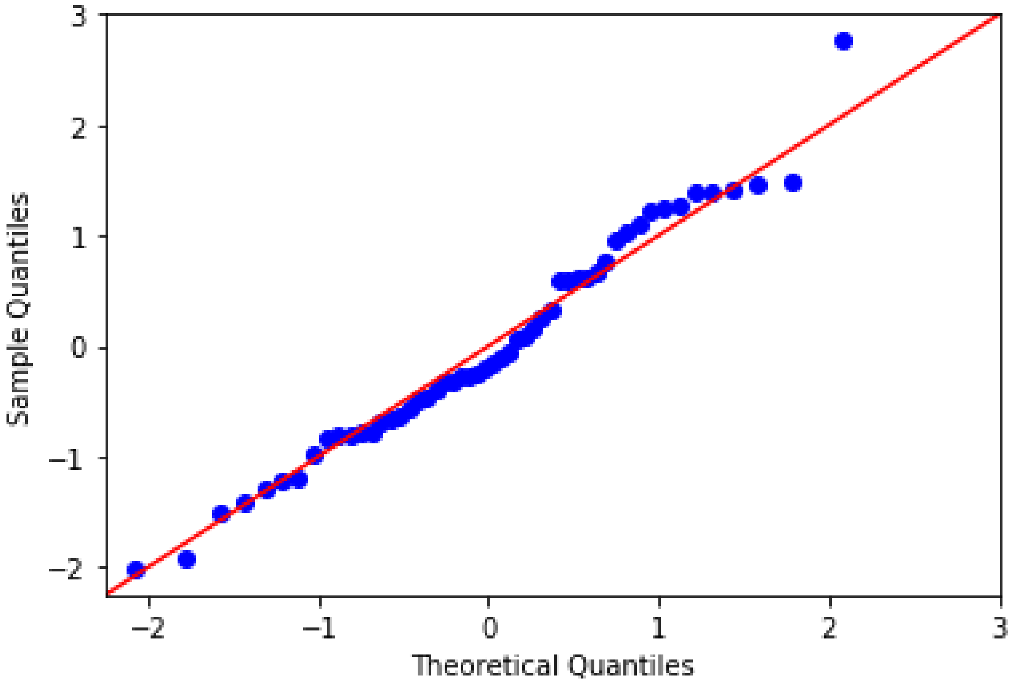

- Distribution : The Jarqu-Bera test probability is 0.664, which means that we cannot reject the null hypothesis that the residuals are not normally distributed. This is also supported by the results of the Omnibus test (probability 0.674) as well as a visual inspection of the residuals in a qq-plot above.

The asssumptions of the models are satisfied with the above mentioned adjustments.

[25]:

```
pw = mod.t_test_pairwise("feed",method="fdr_bh", alpha=0.05)

pw.result_frame
```

[25]:

```
                                                   coef       std err  \
Frugivore-insectivore-Frugivore-folivore   -2.008434e+09  4.278263e+08
Frugivore-omnivore-Frugivore-folivore      -7.767771e+07  3.059119e+08
Gummivore-insectivore-Frugivore-folivore   -1.305064e+09  5.094178e+08
Frugivore-omnivore-Frugivore-insectivore    1.930756e+09  4.246494e+08
Gummivore-insectivore-Frugivore-insectivore 7.033700e+08  5.884314e+08
```

```
Gummivore-insectivore-Frugivore-omnivore    -1.227386e+09  5.067527e+08

                                                   z     P>|z|  \
Frugivore-insectivore-Frugivore-folivore    -4.694508  0.000003
Frugivore-omnivore-Frugivore-folivore       -0.253922  0.799556
Gummivore-insectivore-Frugivore-folivore    -2.561874  0.010411
Frugivore-omnivore-Frugivore-insectivore     4.546707  0.000005
Gummivore-insectivore-Frugivore-insectivore  1.195330  0.231958
Gummivore-insectivore-Frugivore-omnivore    -2.422062  0.015433

                                             Conf. Int. Low  Conf. Int. Upp.  \
Frugivore-insectivore-Frugivore-folivore      -2.846958e+09    -1.169910e+09
Frugivore-omnivore-Frugivore-folivore         -6.772541e+08     5.218987e+08
Gummivore-insectivore-Frugivore-folivore      -2.303505e+09    -3.066235e+08
Frugivore-omnivore-Frugivore-insectivore       1.098459e+09     2.763054e+09
Gummivore-insectivore-Frugivore-insectivore   -4.499343e+08     1.856674e+09
Gummivore-insectivore-Frugivore-omnivore      -2.220603e+09    -2.341694e+08

                                             pvalue-fdr_bh  reject-fdr_bh
Frugivore-insectivore-Frugivore-folivore          0.000016           True
Frugivore-omnivore-Frugivore-folivore             0.799556          False
Gummivore-insectivore-Frugivore-folivore          0.020822           True
Frugivore-omnivore-Frugivore-insectivore          0.000016           True
Gummivore-insectivore-Frugivore-insectivore       0.278350          False
Gummivore-insectivore-Frugivore-omnivore          0.023149           True
```

The pairwise t-test was performed on the result of the ordinary least square regression with the Hochberg-Binyamini p-palue adjustment for multiple hypothesis testing.

1.2.2 Conclusion

The the pairs **frugivo-omnivore/frugivo-folivore** and **gumivore-insectivore/frugivo-insectivore*** are **not statistically significantly different** with the the =0.05. The remaining goups are statistically significantly different.

1.2.3 Shannon diversity

[26]:
```
fig = go.Figure()

x = df2["feed"]

fig.add_trace(go.Box(y=df2["shannon"], x=x, showlegend=False))
```

```
fig.update_layout(template=template, title="Shannon per feed category",␣
↪width=400, height=600)

fig.show()
```

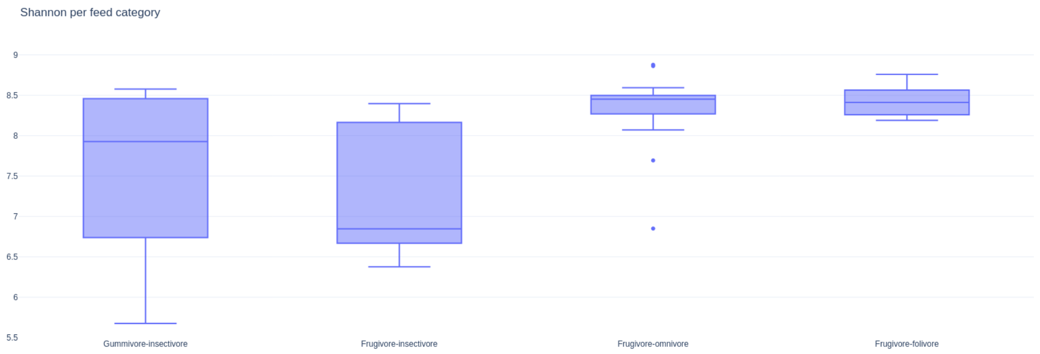

[27]:
```
mod = ols('shannon_boxcox ~ feed', data=df2).fit(cov_type='HC1')

print(mod.summary())
```

```
                            OLS Regression Results
==============================================================================
Dep. Variable:         shannon_boxcox   R-squared:                       0.420
Model:                            OLS   Adj. R-squared:                  0.384
Method:                 Least Squares   F-statistic:                     12.67
Date:                Thu, 25 Mar 2021   Prob (F-statistic):           3.19e-06
Time:                        11:18:25   Log-Likelihood:                -758.88
No. Observations:                  52   AIC:                             1526.
Df Residuals:                      48   BIC:                             1534.
Df Model:                           3
Covariance Type:                  HC1
================================================================================
=================
                                    coef    std err          z      P>|z|
[0.025      0.975]
--------------------------------------------------------------------------------
-----------------
Intercept                      1.757e+06   1.11e+05     15.821      0.000
1.54e+06    1.97e+06
feed[T.Frugivore-insectivore] -1.086e+06   1.97e+05     -5.521      0.000
-1.47e+06      -7e+05
feed[T.Frugivore-omnivore]    -7.361e+04   1.55e+05     -0.476      0.634
-3.77e+05    2.29e+05
feed[T.Gummivore-insectivore] -7.006e+05   2.56e+05     -2.740      0.006
```

```
-1.2e+06   -1.99e+05
==============================================================================
Omnibus:                        0.060   Durbin-Watson:                   1.502
Prob(Omnibus):                  0.970   Jarque-Bera (JB):                0.230
Skew:                           0.056   Prob(JB):                        0.892
Kurtosis:                       2.694   Cond. No.                         6.10
==============================================================================

Warnings:
[1] Standard Errors are heteroscedasticity robust (HC1)
```

[28]:
```
fig = sm.qqplot(mod.resid, fit=True, line="45")
```

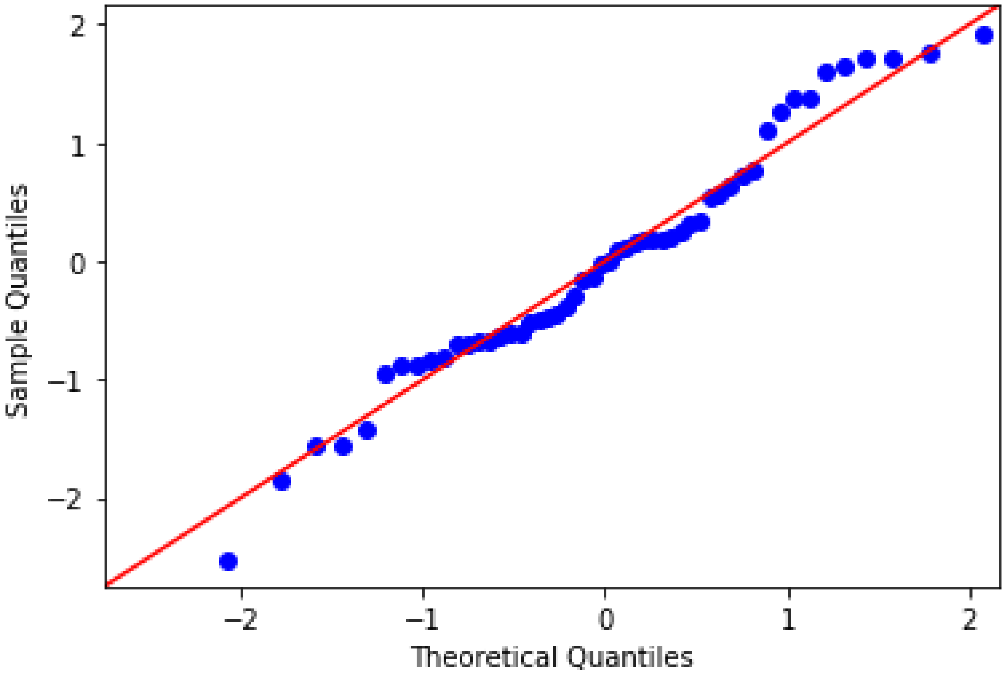

- Distribution : The Jarqu-Bera test probability is 0.892, which means that we cannot reject the null hypothesis that the residuals are not normally distributed. This is also supported by the results of the Omnibus test (probability 0.970) as well as a visual inspection of the residuals in a qq-plot above.

The asssumptions of the models are satisfied with the above mentioned adjustments.

[29]:
```
pw = mod.t_test_pairwise("feed",method="fdr_bh", alpha=0.05)

pw.result_frame
```

[29]:
```
                                                     coef        std err  \
Frugivore-insectivore-Frugivore-folivore    -1.085720e+06  196653.377160
```

```
Frugivore-omnivore-Frugivore-folivore       -7.360826e+04  154586.861791
Gummivore-insectivore-Frugivore-folivore    -7.006441e+05  255743.665868
Frugivore-omnivore-Frugivore-insectivore     1.012112e+06  194680.458493
Gummivore-insectivore-Frugivore-insectivore  3.850758e+05  281794.616188
Gummivore-insectivore-Frugivore-omnivore    -6.270358e+05  254229.724467

                                                    z         P>|z|  \
Frugivore-insectivore-Frugivore-folivore    -5.520983  3.371093e-08
Frugivore-omnivore-Frugivore-folivore       -0.476161  6.339595e-01
Gummivore-insectivore-Frugivore-folivore    -2.739634  6.150759e-03
Frugivore-omnivore-Frugivore-insectivore     5.198835  2.005414e-07
Gummivore-insectivore-Frugivore-insectivore  1.366512  1.717783e-01
Gummivore-insectivore-Frugivore-omnivore    -2.466414  1.364734e-02

                                             Conf. Int. Low  Conf. Int. Upp.  \
Frugivore-insectivore-Frugivore-folivore      -1.471153e+06    -7.002863e+05
Frugivore-omnivore-Frugivore-folivore         -3.765929e+05     2.293764e+05
Gummivore-insectivore-Frugivore-folivore      -1.201892e+06    -1.993957e+05
Frugivore-omnivore-Frugivore-insectivore       6.305449e+05     1.393678e+06
Gummivore-insectivore-Frugivore-insectivore   -1.672315e+05     9.373831e+05
Gummivore-insectivore-Frugivore-omnivore      -1.125317e+06    -1.287547e+05

                                             pvalue-fdr_bh  reject-fdr_bh
Frugivore-insectivore-Frugivore-folivore      2.022656e-07           True
Frugivore-omnivore-Frugivore-folivore         6.339595e-01          False
Gummivore-insectivore-Frugivore-folivore      1.230152e-02           True
Frugivore-omnivore-Frugivore-insectivore      6.016241e-07           True
Gummivore-insectivore-Frugivore-insectivore   2.061339e-01          False
Gummivore-insectivore-Frugivore-omnivore      2.047100e-02           True
```

The pairwise t-test was performed on the result of the ordinary least square regression with the Hochberg-Binyamini p-palue adjustment for multiple hypothesis testing.

1.2.4 Conclusion

The the pairs **frugivo-omnivore/frugivo-folivore** and **gumivore-insectivore/frugivo-insectivore*** are **not statistically significantly different** with the the =0.05. The remaining goups are statistically significantly different.

1.2.5 Pielou evenness

[30]:
```
fig = go.Figure()

x = df2["feed"]
```

```
fig.add_trace(go.Box(y=df2["pielou"], x=x, showlegend=False))

fig.update_layout(template=template, title="Pielou per feed category",␣
 ↪width=400, height=600)

fig.show()
```

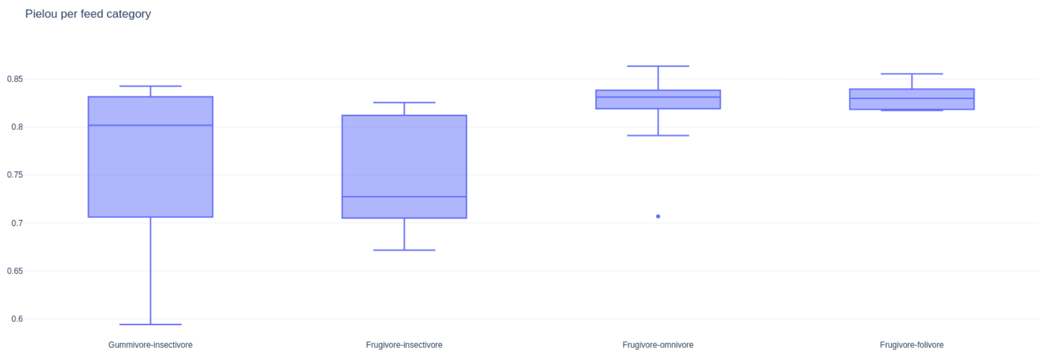

[31]:
```
mod = ols('pielou_boxcox ~ feed', data=df2).fit(cov_type='HC1')

print(mod.summary())
```

```
                            OLS Regression Results
==============================================================================
Dep. Variable:           pielou_boxcox   R-squared:                       0.419
Model:                             OLS   Adj. R-squared:                  0.383
Method:                  Least Squares   F-statistic:                     13.19
Date:                 Thu, 25 Mar 2021   Prob (F-statistic):           2.09e-06
Time:                         11:18:25   Log-Likelihood:                 217.68
No. Observations:                   52   AIC:                            -427.4
Df Residuals:                       48   BIC:                            -419.6
Df Model:                            3
Covariance Type:                   HC1
================================================================================
=================
                                    coef    std err          z      P>|z|
[0.025      0.975]
--------------------------------------------------------------------------------
-----------------
Intercept                        -0.0796      0.001    -98.055      0.000
-0.081      -0.078
feed[T.Frugivore-insectivore]    -0.0076      0.001     -5.640      0.000
-0.010      -0.005
```

```
feed[T.Frugivore-omnivore]       -0.0006      0.001     -0.507      0.612
-0.003       0.002
feed[T.Gummivore-insectivore]    -0.0048      0.002     -2.702      0.007
-0.008      -0.001
==============================================================================
Omnibus:                        0.102   Durbin-Watson:                   1.571
Prob(Omnibus):                  0.950   Jarque-Bera (JB):                0.060
Skew:                          -0.065   Prob(JB):                        0.971
Kurtosis:                       2.898   Cond. No.                         6.10
==============================================================================

Warnings:
[1] Standard Errors are heteroscedasticity robust (HC1)
```

[32]:
```
fig = sm.qqplot(mod.resid, fit=True, line="45")
```

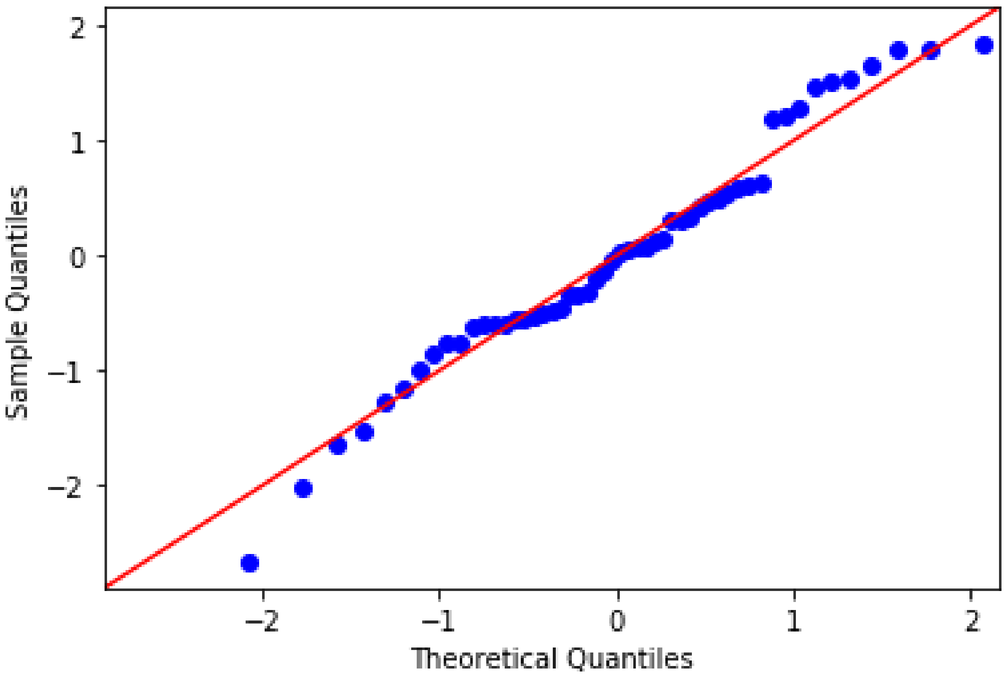

- Distribution : The Jarqu-Bera test probability is 0.971, which means that we cannot reject the null hypothesis that the residuals are not normally distributed. This is also supported by the results of the Omnibus test (probability 0.950) as well as a visual inspection of the residuals in a qq-plot above.

The asssumptions of the models are satisfied with the above mentioned adjustments.

[33]:
```
pw = mod.t_test_pairwise("feed",method="fdr_bh", alpha=0.05)

pw.result_frame
```

[33]:
```
                                                coef   std err         z  \
Frugivore-insectivore-Frugivore-folivore    -0.007630  0.001353 -5.640258
Frugivore-omnivore-Frugivore-folivore       -0.000572  0.001127 -0.507065
Gummivore-insectivore-Frugivore-folivore    -0.004824  0.001785 -2.702483
Frugivore-omnivore-Frugivore-insectivore     0.007058  0.001335  5.286880
Gummivore-insectivore-Frugivore-insectivore  0.002806  0.001923  1.458927
Gummivore-insectivore-Frugivore-omnivore    -0.004253  0.001772 -2.400249

                                                    P>|z|  Conf. Int. Low  \
Frugivore-insectivore-Frugivore-folivore     1.697959e-08       -0.010281
Frugivore-omnivore-Frugivore-folivore        6.121093e-01       -0.002781
Gummivore-insectivore-Frugivore-folivore     6.882371e-03       -0.008323
Frugivore-omnivore-Frugivore-insectivore     1.244204e-07        0.004442
Gummivore-insectivore-Frugivore-insectivore  1.445853e-01       -0.000964
Gummivore-insectivore-Frugivore-omnivore     1.638391e-02       -0.007725

                                             Conf. Int. Upp.  pvalue-fdr_bh  \
Frugivore-insectivore-Frugivore-folivore           -0.004978   1.018776e-07
Frugivore-omnivore-Frugivore-folivore               0.001638   6.121093e-01
Gummivore-insectivore-Frugivore-folivore           -0.001325   1.376474e-02
Frugivore-omnivore-Frugivore-insectivore            0.009675   3.732613e-07
Gummivore-insectivore-Frugivore-insectivore         0.006575   1.735023e-01
Gummivore-insectivore-Frugivore-omnivore           -0.000780   2.457587e-02

                                             reject-fdr_bh
Frugivore-insectivore-Frugivore-folivore              True
Frugivore-omnivore-Frugivore-folivore                False
Gummivore-insectivore-Frugivore-folivore              True
Frugivore-omnivore-Frugivore-insectivore              True
Gummivore-insectivore-Frugivore-insectivore          False
Gummivore-insectivore-Frugivore-omnivore              True
```

The pairwise t-test was performed on the result of the ordinary least square regression with the Hochberg-Binyamini p-palue adjustment for multiple hypothesis testing.

1.2.6 Conclusion

The the pairs **frugivo-omnivore/frugivo-folivore** and **gumivore-insectivore/frugivo-insectivore*** are **not statistically significantly different** with the the =0.05. The remaining goups are statistically significantly different.

Supplementary S2

March 25, 2021

1 Ancom statistics

The following statistical analysis aims to identify groups that are statistically significantly different between the given categories. Data is loaded and normalized in in the same way as for the main analysis including using the same seed for normalization. This produces an identical outcome.

[1]:
```
import sys
import copy

sys.path.append("/home/user/Programs/seqDataClass")

import pandas as pd
import numpy as np
import scipy as sp
import random

import plotly.express as px
import plotly.graph_objs as go
from plotly.subplots import make_subplots

from seqDataClass import seqObject
#import seqDataClass

from skbio.stats.composition import ancom, multiplicative_replacement

idx = pd.IndexSlice

template = 'plotly_white'

def ancom_plot(ancom_df, percentile_df, width = 1000, height = 500, ngroups =␣
 ↪3):
    fig = go.Figure()

    names = []
    for entry in ancom_df.sort_values(by="W", ascending=False)[0:ngroups].index:
        names.append(entry)
```

```
    for category in list(set(percentile_df.columns.
↪to_frame(index=False)["Group"])):
        fig.add_trace(go.Box(x=names,
                             y=[],
                             name=category))

        fig.update_traces(selector = dict(name=category),
                          lowerfence = percentile_df.loc[names,idx[5.0,␣
↪category]],
                          q1 = percentile_df.loc[names,idx[25.0, category]],
                          median = percentile_df.loc[names,idx[50.0, category]],
                          q3= percentile_df.loc[names,idx[75.0, category]],
                          upperfence= percentile_df.loc[names,idx[95.0,␣
↪category]]
                         )
    fig.update_layout(
        boxmode='group', # group together boxes of the different traces for␣
↪each value of x
        width = width,
        height = height,
        template = "plotly_white"
    )

    fig.show()

def ancom_dataprep(seq, tax_level):

    class_data = seq.data.sum(axis=0, level=tax_level)

    df1 = class_data.transpose()

    df2 = pd.DataFrame(multiplicative_replacement(df1))

    df1idx = df1.index.to_frame(index=False)
    df1col = df1.columns.to_frame(index=False)

    df2.columns = df1col[tax_level]

    return df2, df1idx
```

```
/home/user/Programs/anaconda3/envs/bioconda/lib/python3.6/site-
packages/skbio/util/_testing.py:16: FutureWarning:

pandas.util.testing is deprecated. Use the functions in the public API at
pandas.testing instead.
```

[2]:
```
filepath = "/home/user/qiime/monkeys/"

seq1 = seqObject(mappingFile = filepath+"mapping file_monkeys_simplified.csv",
                 taxonomyFile = filepath+"dada2_taxonomy_updated.csv",
                 featureFile = filepath+"dada2_feature_table_nochim.csv",
                 mappingSep = ";",
                 taxonomySep = ",",
                 featureSep = ",",
                 sampleNamesColumn = "sample-id",
                 #featureFormat = "dada2",
                 #taxonomyFormat = "dada2"
                 )
```

```
Initializing data loading.
Loading mapping file
Loading taxonomy
Recognizing a DADA2 format.
Matching features between feature file and taxonomy file.
Features match.
```

[3]:
```
seq1.extract_rep_seq(filename=filepath+"rep_seq.fasta", renameFeatures=True)
```

[4]:
```
seq1.rarefy_to_even_depth(seqDepth=42134, seed=123)
seq1.extract_features(tax_level="feature-id",
                      file_name="monkeys_otu_table_normalized.csv",
                      show_output=False)
```

```
Removing 2513 features/OTUs that no longer appear in any sample.
```

[4]:
```
'Features extracted in the file monkeys_otu_table_normalized.csv'
```

1.1 Difference in orders between new and old world primates

The difference between the **taxonomic phyla groups** between the old and the new world primates was calculated on = 0.05 using a Holm-Bonferroni method of adjustment for multiple hypothesis testing.

1.1.1 Phylum level

[5]:
```
df1, df1idx =  ancom_dataprep(seq  = seq1, tax_level = "Phylum")

ancom_df, percentile_df = ancom(df1,
                                grouping=df1idx["sub-order"],
                                significance_test=sp.stats.f_oneway,
                                alpha=0.05,     # Can and should perhaps be␣
↪adjusted  to a lower value (still works with alpha 0.001)
```

```
                                ␣
↪multiple_comparisons_correction='holm-bonferroni',
                                percentiles=[5.0, 25.0, 50.0, 75.0, 95.0])

ancom_df.sort_values(by="W", ascending=False)[0:5]
```

[5]:

```
                     W  Reject null hypothesis
Phylum
Actinobacteriota    13                    True
Campilobacterota    12                    True
Proteobacteria       8                   False
Verrucomicrobiota    8                   False
Bdellovibrionota     7                   False
```

The phyla Actinobacteriota and Campilobacterota are significantly different between the new world and old world monkeys. This is visualized in the figure below.

[6]:

```
ancom_plot(ancom_df, percentile_df, ngroups=2, width=500)
```

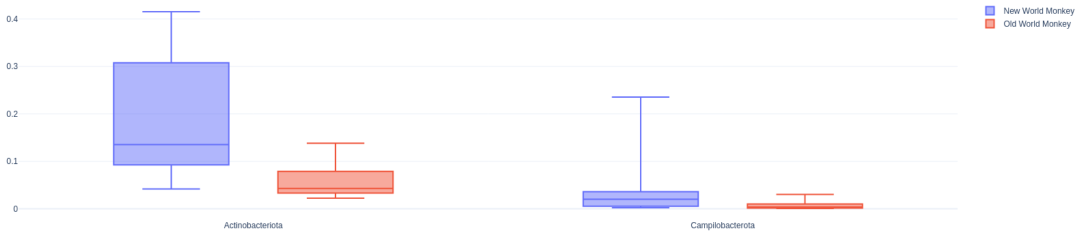

1.1.2 Family level

The difference between the **taxonomic family groups** between the old and the new world primates was calculated on $= 0.05$ using a Holm-Bonferroni method of adjustment for multiple hypothesis testing.

[7]:

```
df1, df1idx =  ancom_dataprep(seq  = seq1, tax_level = "Family")

ancom_df, percentile_df = ancom(df1,
                                grouping=df1idx["sub-order"],
                                significance_test=sp.stats.f_oneway,
                                alpha=0.05,     # Can and should perhaps be␣
↪adjusted  to a lower value (still works with alpha 0.001)
                                ␣
↪multiple_comparisons_correction='holm-bonferroni',
```

```
                                percentiles=[5.0, 25.0, 50.0, 75.0, 95.0])

ancom_df.sort_values(by="W", ascending=False)[0:5]
```

```
/home/user/Programs/anaconda3/envs/bioconda/lib/python3.6/site-
packages/scipy/stats/stats.py:3349: RuntimeWarning:

invalid value encountered in double_scalars
```

[7]:

Family	W	Reject null hypothesis
Bifidobacteriaceae	139	True
Oscillospiraceae	113	False
Coriobacteriaceae	109	False
Ruminococcaceae	107	False
[Eubacterium] coprostanoligenes group	106	False

Only the group Bifidobacteriaceae is significantly different between the new world and old world monkeys. This is visualized below.

[8]:
```
ancom_plot(ancom_df, percentile_df, ngroups=1, width=500)
```

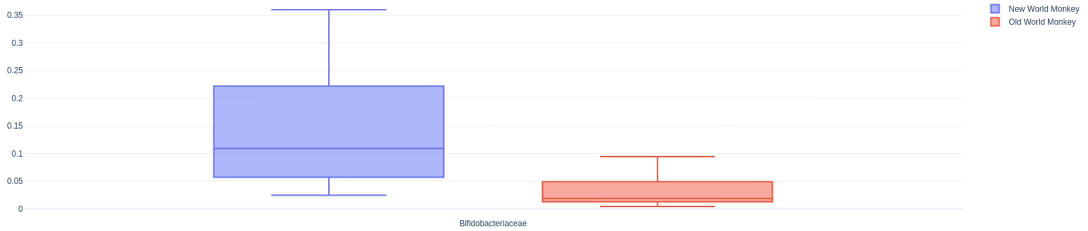

1.1.3 Species level

The difference between the **bacterial species** between the old and the new world primates was calculated on = 0.05 using a Holm-Bonferroni method of adjustment for multiple hypothesis testing.

[9]:
```
df1, df1idx =  ancom_dataprep(seq  = seq1, tax_level = "Species")

ancom_df, percentile_df = ancom(df1,
                                grouping=df1idx["sub-order"],
                                significance_test=sp.stats.f_oneway,
```

```
                                alpha=0.05,     # Can and should perhaps be␣
↪adjusted  to a lower value (still works with alpha 0.001)
                                ␣
↪multiple_comparisons_correction='holm-bonferroni',
                                percentiles=[5.0, 25.0, 50.0, 75.0, 95.0])

ancom_df.sort_values(by="W", ascending=False)[0:10]
```

```
/home/user/Programs/anaconda3/envs/bioconda/lib/python3.6/site-
packages/scipy/stats/stats.py:3349: RuntimeWarning:

invalid value encountered in double_scalars
```

[9]:

	W	Reject null hypothesis
Species		
callitrichos	38	True
parmae 1	38	True
saguini	34	True
biavatii	34	True
vansinderenii	34	True
aerophilum	34	True
unclassified II	33	True
sp. I	30	True
unclassified III	26	False
ramosum	24	False

[10]:

```
ancom_plot(ancom_df, percentile_df, ngroups=8, width=1000)
```

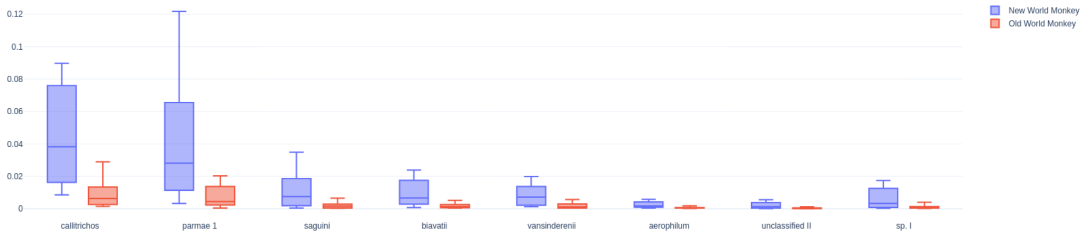

1.2 Differences due to feed classification

1.2.1 Phylum level

The difference between the **taxonomic phylum groups** feed types was calculated on $= 0.05$ using a Holm-Bonferroni method of adjustment for multiple hypothesis testing.

[11]:
```
df1, df1idx =  ancom_dataprep(seq  = seq1, tax_level = "Phylum")

ancom_df, percentile_df = ancom(df1,
                                grouping=df1idx["feed"],
                                significance_test=sp.stats.f_oneway,
                                alpha=0.05,     # Can and should perhaps be␣
 ↪adjusted  to a lower value (still works with alpha 0.001)
                                                                                    ␣
 ↪multiple_comparisons_correction='holm-bonferroni',
                                percentiles=[5.0, 25.0, 50.0, 75.0, 95.0])

ancom_df.sort_values(by="W", ascending=False)[0:5]
```

[11]:

Phylum	W	Reject null hypothesis
Actinobacteriota	12	True
Proteobacteria	8	True
Campilobacterota	7	True
Spirochaetota	6	False
Verrucomicrobiota	6	False

The phyla Actinobacteria, Proteobacteria, Campilobacterota are significantly different between the feed categories. This is visualized bellow.

[12]:
```
ancom_plot(ancom_df, percentile_df, ngroups=3, width=500)
```

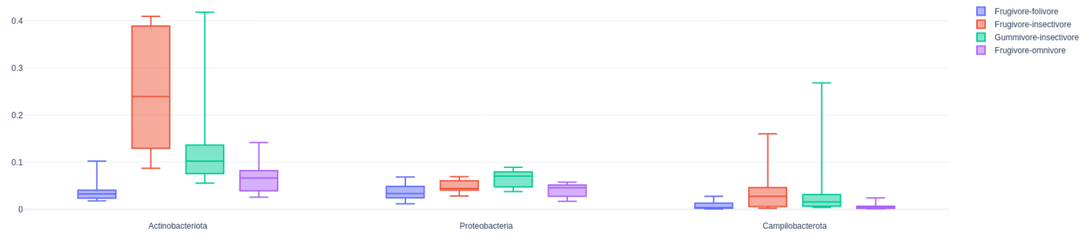

1.2.2 Family level

The difference between the **taxonomic family groups** feed types was calculated on $= 0.05$ using a Holm-Bonferroni method of adjustment for multiple hypothesis testing.

[13]:
```
#tax_level = "Family"
#class_data = seq1.data.sum(axis=0, level=tax_level)
df1, df1idx =  ancom_dataprep(seq  = seq1, tax_level = "Family")

ancom_df, percentile_df = ancom(df1,
                                grouping=df1idx["feed"],
                                significance_test=sp.stats.f_oneway,
                                alpha=0.05,     # Can and should perhaps be␣
 ↪adjusted  to a lower value (still works with alpha 0.001)
                                                            ␣
 ↪multiple_comparisons_correction='holm-bonferroni',
                                percentiles=[5.0, 25.0, 50.0, 75.0, 95.0])

ancom_df.sort_values(by="W", ascending=False)[0:5]
```

```
/home/user/Programs/anaconda3/envs/bioconda/lib/python3.6/site-
packages/scipy/stats/stats.py:3349: RuntimeWarning:

invalid value encountered in double_scalars
```

[13]:

Family	W	Reject null hypothesis
Bifidobacteriaceae	140	True
Coriobacteriaceae	114	False
Ruminococcaceae	100	False
Lachnospiraceae	96	False
Spirochaetaceae	95	False

[14]:
```
ancom_plot(ancom_df, percentile_df, ngroups=1, width=500)
```

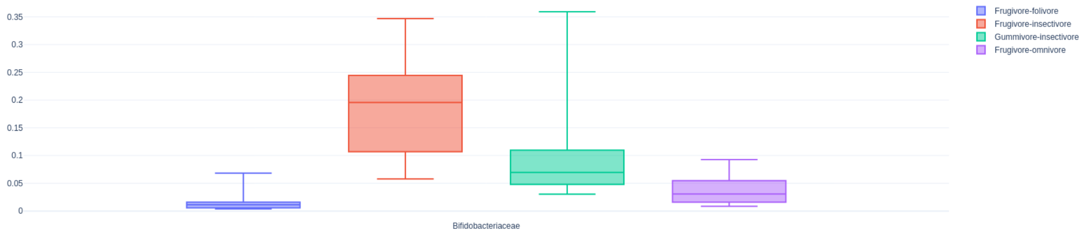

Supplementary S3

DADA2 analysis

```r
library(dada2); packageVersion("dada2")
```

```
## Loading required package: Rcpp
```

```
## [1] '1.16.0'
```

```r
library("seqinr")
```

```r
my_path = "/home/user/qiime/monkeys/raw"
my_pat_root = "/home/user/qiime/monkeys"
list.files (path = my_path)
```

```
##   [1] "filtered"          "PR1.raw_1.fq.gz"  "PR1.raw_2.fq.gz"
##   [4] "PR10.raw_1.fq.gz" "PR10.raw_2.fq.gz" "PR11.raw_1.fq.gz"
##   [7] "PR11.raw_2.fq.gz" "PR15.raw_1.fq.gz" "PR15.raw_2.fq.gz"
##  [10] "PR16.raw_1.fq.gz" "PR16.raw_2.fq.gz" "PR17.raw_1.fq.gz"
##  [13] "PR17.raw_2.fq.gz" "PR18.raw_1.fq.gz" "PR18.raw_2.fq.gz"
##  [16] "PR19.raw_1.fq.gz" "PR19.raw_2.fq.gz" "PR2.raw_1.fq.gz"
##  [19] "PR2.raw_2.fq.gz"  "PR20.raw_1.fq.gz" "PR20.raw_2.fq.gz"
##  [22] "PR21.raw_1.fq.gz" "PR21.raw_2.fq.gz" "PR22.raw_1.fq.gz"
##  [25] "PR22.raw_2.fq.gz" "PR23.raw_1.fq.gz" "PR23.raw_2.fq.gz"
##  [28] "PR24.raw_1.fq.gz" "PR24.raw_2.fq.gz" "PR26.raw_1.fq.gz"
##  [31] "PR26.raw_2.fq.gz" "PR27.raw_1.fq.gz" "PR27.raw_2.fq.gz"
##  [34] "PR28.raw_1.fq.gz" "PR28.raw_2.fq.gz" "PR29.raw_1.fq.gz"
##  [37] "PR29.raw_2.fq.gz" "PR3.raw_1.fq.gz"  "PR3.raw_2.fq.gz"
##  [40] "PR30.raw_1.fq.gz" "PR30.raw_2.fq.gz" "PR31.raw_1.fq.gz"
##  [43] "PR31.raw_2.fq.gz" "PR32.raw_1.fq.gz" "PR32.raw_2.fq.gz"
##  [46] "PR33.raw_1.fq.gz" "PR33.raw_2.fq.gz" "PR34.raw_1.fq.gz"
##  [49] "PR34.raw_2.fq.gz" "PR35.raw_1.fq.gz" "PR35.raw_2.fq.gz"
##  [52] "PR36.raw_1.fq.gz" "PR36.raw_2.fq.gz" "PR37.raw_1.fq.gz"
##  [55] "PR37.raw_2.fq.gz" "PR38.raw_1.fq.gz" "PR38.raw_2.fq.gz"
##  [58] "PR39.raw_1.fq.gz" "PR39.raw_2.fq.gz" "PR4.raw_1.fq.gz"
##  [61] "PR4.raw_2.fq.gz"  "PR40.raw_1.fq.gz" "PR40.raw_2.fq.gz"
##  [64] "PR41.raw_1.fq.gz" "PR41.raw_2.fq.gz" "PR42.raw_1.fq.gz"
##  [67] "PR42.raw_2.fq.gz" "PR43.raw_1.fq.gz" "PR43.raw_2.fq.gz"
##  [70] "PR44.raw_1.fq.gz" "PR44.raw_2.fq.gz" "PR45.raw_1.fq.gz"
##  [73] "PR45.raw_2.fq.gz" "PR46.raw_1.fq.gz" "PR46.raw_2.fq.gz"
##  [76] "PR47.raw_1.fq.gz" "PR47.raw_2.fq.gz" "PR5.raw_1.fq.gz"
##  [79] "PR5.raw_2.fq.gz"  "PR51.raw_1.fq.gz" "PR51.raw_2.fq.gz"
##  [82] "PR52.raw_1.fq.gz" "PR52.raw_2.fq.gz" "PR55.raw_1.fq.gz"
##  [85] "PR55.raw_2.fq.gz" "PR56.raw_1.fq.gz" "PR56.raw_2.fq.gz"
##  [88] "PR57.raw_1.fq.gz" "PR57.raw_2.fq.gz" "PR58.raw_1.fq.gz"
##  [91] "PR58.raw_2.fq.gz" "PR59.raw_1.fq.gz" "PR59.raw_2.fq.gz"
##  [94] "PR6.raw_1.fq.gz"  "PR6.raw_2.fq.gz"  "PR60.raw_1.fq.gz"
##  [97] "PR60.raw_2.fq.gz" "PR61.raw_1.fq.gz" "PR61.raw_2.fq.gz"
## [100] "PR7.raw_1.fq.gz"  "PR7.raw_2.fq.gz"  "PR8.raw_1.fq.gz"
## [103] "PR8.raw_2.fq.gz"  "PR9.raw_1.fq.gz"  "PR9.raw_2.fq.gz"
```

```r
list.files(my_path, pattern = "_1.fq.gz", full.names = TRUE)
```

```
##  [1] "/home/user/qiime/monkeys/raw/PR1.raw_1.fq.gz"
##  [2] "/home/user/qiime/monkeys/raw/PR10.raw_1.fq.gz"
##  [3] "/home/user/qiime/monkeys/raw/PR11.raw_1.fq.gz"
##  [4] "/home/user/qiime/monkeys/raw/PR15.raw_1.fq.gz"
##  [5] "/home/user/qiime/monkeys/raw/PR16.raw_1.fq.gz"
##  [6] "/home/user/qiime/monkeys/raw/PR17.raw_1.fq.gz"
##  [7] "/home/user/qiime/monkeys/raw/PR18.raw_1.fq.gz"
##  [8] "/home/user/qiime/monkeys/raw/PR19.raw_1.fq.gz"
##  [9] "/home/user/qiime/monkeys/raw/PR2.raw_1.fq.gz"
## [10] "/home/user/qiime/monkeys/raw/PR20.raw_1.fq.gz"
## [11] "/home/user/qiime/monkeys/raw/PR21.raw_1.fq.gz"
## [12] "/home/user/qiime/monkeys/raw/PR22.raw_1.fq.gz"
## [13] "/home/user/qiime/monkeys/raw/PR23.raw_1.fq.gz"
## [14] "/home/user/qiime/monkeys/raw/PR24.raw_1.fq.gz"
## [15] "/home/user/qiime/monkeys/raw/PR26.raw_1.fq.gz"
## [16] "/home/user/qiime/monkeys/raw/PR27.raw_1.fq.gz"
## [17] "/home/user/qiime/monkeys/raw/PR28.raw_1.fq.gz"
## [18] "/home/user/qiime/monkeys/raw/PR29.raw_1.fq.gz"
## [19] "/home/user/qiime/monkeys/raw/PR3.raw_1.fq.gz"
## [20] "/home/user/qiime/monkeys/raw/PR30.raw_1.fq.gz"
## [21] "/home/user/qiime/monkeys/raw/PR31.raw_1.fq.gz"
## [22] "/home/user/qiime/monkeys/raw/PR32.raw_1.fq.gz"
## [23] "/home/user/qiime/monkeys/raw/PR33.raw_1.fq.gz"
## [24] "/home/user/qiime/monkeys/raw/PR34.raw_1.fq.gz"
## [25] "/home/user/qiime/monkeys/raw/PR35.raw_1.fq.gz"
## [26] "/home/user/qiime/monkeys/raw/PR36.raw_1.fq.gz"
## [27] "/home/user/qiime/monkeys/raw/PR37.raw_1.fq.gz"
## [28] "/home/user/qiime/monkeys/raw/PR38.raw_1.fq.gz"
## [29] "/home/user/qiime/monkeys/raw/PR39.raw_1.fq.gz"
## [30] "/home/user/qiime/monkeys/raw/PR4.raw_1.fq.gz"
## [31] "/home/user/qiime/monkeys/raw/PR40.raw_1.fq.gz"
## [32] "/home/user/qiime/monkeys/raw/PR41.raw_1.fq.gz"
## [33] "/home/user/qiime/monkeys/raw/PR42.raw_1.fq.gz"
## [34] "/home/user/qiime/monkeys/raw/PR43.raw_1.fq.gz"
## [35] "/home/user/qiime/monkeys/raw/PR44.raw_1.fq.gz"
## [36] "/home/user/qiime/monkeys/raw/PR45.raw_1.fq.gz"
## [37] "/home/user/qiime/monkeys/raw/PR46.raw_1.fq.gz"
## [38] "/home/user/qiime/monkeys/raw/PR47.raw_1.fq.gz"
## [39] "/home/user/qiime/monkeys/raw/PR5.raw_1.fq.gz"
## [40] "/home/user/qiime/monkeys/raw/PR51.raw_1.fq.gz"
## [41] "/home/user/qiime/monkeys/raw/PR52.raw_1.fq.gz"
## [42] "/home/user/qiime/monkeys/raw/PR55.raw_1.fq.gz"
## [43] "/home/user/qiime/monkeys/raw/PR56.raw_1.fq.gz"
## [44] "/home/user/qiime/monkeys/raw/PR57.raw_1.fq.gz"
## [45] "/home/user/qiime/monkeys/raw/PR58.raw_1.fq.gz"
## [46] "/home/user/qiime/monkeys/raw/PR59.raw_1.fq.gz"
## [47] "/home/user/qiime/monkeys/raw/PR6.raw_1.fq.gz"
## [48] "/home/user/qiime/monkeys/raw/PR60.raw_1.fq.gz"
## [49] "/home/user/qiime/monkeys/raw/PR61.raw_1.fq.gz"
## [50] "/home/user/qiime/monkeys/raw/PR7.raw_1.fq.gz"
## [51] "/home/user/qiime/monkeys/raw/PR8.raw_1.fq.gz"
## [52] "/home/user/qiime/monkeys/raw/PR9.raw_1.fq.gz"
```

```r
list.files(my_path, pattern = "_2.fq.gz", full.names = TRUE)
```

```
##  [1] "/home/user/qiime/monkeys/raw/PR1.raw_2.fq.gz"
##  [2] "/home/user/qiime/monkeys/raw/PR10.raw_2.fq.gz"
##  [3] "/home/user/qiime/monkeys/raw/PR11.raw_2.fq.gz"
##  [4] "/home/user/qiime/monkeys/raw/PR15.raw_2.fq.gz"
##  [5] "/home/user/qiime/monkeys/raw/PR16.raw_2.fq.gz"
##  [6] "/home/user/qiime/monkeys/raw/PR17.raw_2.fq.gz"
##  [7] "/home/user/qiime/monkeys/raw/PR18.raw_2.fq.gz"
##  [8] "/home/user/qiime/monkeys/raw/PR19.raw_2.fq.gz"
##  [9] "/home/user/qiime/monkeys/raw/PR2.raw_2.fq.gz"
## [10] "/home/user/qiime/monkeys/raw/PR20.raw_2.fq.gz"
## [11] "/home/user/qiime/monkeys/raw/PR21.raw_2.fq.gz"
## [12] "/home/user/qiime/monkeys/raw/PR22.raw_2.fq.gz"
## [13] "/home/user/qiime/monkeys/raw/PR23.raw_2.fq.gz"
## [14] "/home/user/qiime/monkeys/raw/PR24.raw_2.fq.gz"
## [15] "/home/user/qiime/monkeys/raw/PR26.raw_2.fq.gz"
## [16] "/home/user/qiime/monkeys/raw/PR27.raw_2.fq.gz"
## [17] "/home/user/qiime/monkeys/raw/PR28.raw_2.fq.gz"
## [18] "/home/user/qiime/monkeys/raw/PR29.raw_2.fq.gz"
## [19] "/home/user/qiime/monkeys/raw/PR3.raw_2.fq.gz"
## [20] "/home/user/qiime/monkeys/raw/PR30.raw_2.fq.gz"
## [21] "/home/user/qiime/monkeys/raw/PR31.raw_2.fq.gz"
## [22] "/home/user/qiime/monkeys/raw/PR32.raw_2.fq.gz"
## [23] "/home/user/qiime/monkeys/raw/PR33.raw_2.fq.gz"
## [24] "/home/user/qiime/monkeys/raw/PR34.raw_2.fq.gz"
## [25] "/home/user/qiime/monkeys/raw/PR35.raw_2.fq.gz"
## [26] "/home/user/qiime/monkeys/raw/PR36.raw_2.fq.gz"
## [27] "/home/user/qiime/monkeys/raw/PR37.raw_2.fq.gz"
## [28] "/home/user/qiime/monkeys/raw/PR38.raw_2.fq.gz"
## [29] "/home/user/qiime/monkeys/raw/PR39.raw_2.fq.gz"
## [30] "/home/user/qiime/monkeys/raw/PR4.raw_2.fq.gz"
## [31] "/home/user/qiime/monkeys/raw/PR40.raw_2.fq.gz"
## [32] "/home/user/qiime/monkeys/raw/PR41.raw_2.fq.gz"
## [33] "/home/user/qiime/monkeys/raw/PR42.raw_2.fq.gz"
## [34] "/home/user/qiime/monkeys/raw/PR43.raw_2.fq.gz"
## [35] "/home/user/qiime/monkeys/raw/PR44.raw_2.fq.gz"
## [36] "/home/user/qiime/monkeys/raw/PR45.raw_2.fq.gz"
## [37] "/home/user/qiime/monkeys/raw/PR46.raw_2.fq.gz"
## [38] "/home/user/qiime/monkeys/raw/PR47.raw_2.fq.gz"
## [39] "/home/user/qiime/monkeys/raw/PR5.raw_2.fq.gz"
## [40] "/home/user/qiime/monkeys/raw/PR51.raw_2.fq.gz"
## [41] "/home/user/qiime/monkeys/raw/PR52.raw_2.fq.gz"
## [42] "/home/user/qiime/monkeys/raw/PR55.raw_2.fq.gz"
## [43] "/home/user/qiime/monkeys/raw/PR56.raw_2.fq.gz"
## [44] "/home/user/qiime/monkeys/raw/PR57.raw_2.fq.gz"
## [45] "/home/user/qiime/monkeys/raw/PR58.raw_2.fq.gz"
## [46] "/home/user/qiime/monkeys/raw/PR59.raw_2.fq.gz"
## [47] "/home/user/qiime/monkeys/raw/PR6.raw_2.fq.gz"
## [48] "/home/user/qiime/monkeys/raw/PR60.raw_2.fq.gz"
## [49] "/home/user/qiime/monkeys/raw/PR61.raw_2.fq.gz"
## [50] "/home/user/qiime/monkeys/raw/PR7.raw_2.fq.gz"
## [51] "/home/user/qiime/monkeys/raw/PR8.raw_2.fq.gz"
## [52] "/home/user/qiime/monkeys/raw/PR9.raw_2.fq.gz"
```

```
fnFs = list.files(my_path, pattern = "_1.fq.gz", full.names = TRUE)
fnRs = list.files(my_path, pattern = "_2.fq.gz", full.names = TRUE)

fnFs = sort(fnFs)
fnRs = sort(fnRs)

sample.names = as.character(sapply(fnFs, function(x) unlist(strsplit(basename(x), split = ".raw_"))[1] )

# Place filtered files in filtered/ subdirectory
filtFs <- file.path(my_path, "filtered", paste0(sample.names, "_F_filt.fastq.gz"))
filtRs <- file.path(my_path, "filtered", paste0(sample.names, "_R_filt.fastq.gz"))
names(filtFs) <- sample.names
names(filtRs) <- sample.names

out = filterAndTrim(fnFs,
                    filtFs,
                    fnRs,
                    filtRs,
                    trimLeft = 22,
                    trimRight = 0,
                    maxN = 0,
                    maxEE = c(2,2),
                    truncQ = 2,
                    rm.phix = TRUE,
                    compress = TRUE,
                    multithread = TRUE)

#options(repr.plot.width = 20,repr.plot.height = 10)
#plotQualityProfile(fnFs[1:3])

#list.files (path = my_path)

filtFs = file.path(my_path, "filtered", paste0(sample.names, "_F_filt.fastq.gz"))
filtRs <- file.path(my_path, "filtered", paste0(sample.names, "_R_filt.fastq.gz"))
names(filtFs) <- sample.names
names(filtRs) <- sample.names
```

```
errF = learnErrors(fls = filtFs, nbases = 1e8, multithread = TRUE)
```

```
## 101178355 total bases in 443815 reads from 4 samples will be used for learning the error rates.
```

```
errR = learnErrors(fls = filtRs, nbases = 1e8, multithread = TRUE)
```

```
## 101176227 total bases in 443815 reads from 4 samples will be used for learning the error rates.
```

```
dadaFs = dada(filtFs, errF, multithread = TRUE)
```

```
## Sample 1 - 50997 reads in 15454 unique sequences.
## Sample 2 - 129948 reads in 35053 unique sequences.
## Sample 3 - 133768 reads in 33668 unique sequences.
## Sample 4 - 129102 reads in 30460 unique sequences.
## Sample 5 - 121156 reads in 30653 unique sequences.
## Sample 6 - 133796 reads in 33167 unique sequences.
## Sample 7 - 122418 reads in 37374 unique sequences.
## Sample 8 - 120891 reads in 37806 unique sequences.
## Sample 9 - 72059 reads in 22197 unique sequences.
```

```
## Sample 10 - 138862 reads in 47613 unique sequences.
## Sample 11 - 136191 reads in 44815 unique sequences.
## Sample 12 - 132129 reads in 44895 unique sequences.
## Sample 13 - 130915 reads in 46145 unique sequences.
## Sample 14 - 122136 reads in 43307 unique sequences.
## Sample 15 - 123426 reads in 39206 unique sequences.
## Sample 16 - 130415 reads in 42822 unique sequences.
## Sample 17 - 132945 reads in 42319 unique sequences.
## Sample 18 - 131107 reads in 43608 unique sequences.
## Sample 19 - 99749 reads in 25495 unique sequences.
## Sample 20 - 132778 reads in 42095 unique sequences.
## Sample 21 - 130653 reads in 41418 unique sequences.
## Sample 22 - 134573 reads in 40954 unique sequences.
## Sample 23 - 126227 reads in 42355 unique sequences.
## Sample 24 - 138808 reads in 41459 unique sequences.
## Sample 25 - 123638 reads in 40562 unique sequences.
## Sample 26 - 133383 reads in 44497 unique sequences.
## Sample 27 - 127008 reads in 43970 unique sequences.
## Sample 28 - 130717 reads in 43284 unique sequences.
## Sample 29 - 126983 reads in 41508 unique sequences.
## Sample 30 - 125192 reads in 31746 unique sequences.
## Sample 31 - 125192 reads in 39747 unique sequences.
## Sample 32 - 123257 reads in 40176 unique sequences.
## Sample 33 - 136461 reads in 41584 unique sequences.
## Sample 34 - 122183 reads in 37618 unique sequences.
## Sample 35 - 135949 reads in 45810 unique sequences.
## Sample 36 - 130411 reads in 45069 unique sequences.
## Sample 37 - 138592 reads in 45044 unique sequences.
## Sample 38 - 134629 reads in 46760 unique sequences.
## Sample 39 - 129737 reads in 36715 unique sequences.
## Sample 40 - 127445 reads in 44164 unique sequences.
## Sample 41 - 123235 reads in 45770 unique sequences.
## Sample 42 - 126827 reads in 44508 unique sequences.
## Sample 43 - 129972 reads in 43550 unique sequences.
## Sample 44 - 138434 reads in 44457 unique sequences.
## Sample 45 - 121764 reads in 42108 unique sequences.
## Sample 46 - 125875 reads in 41283 unique sequences.
## Sample 47 - 135450 reads in 37224 unique sequences.
## Sample 48 - 121771 reads in 39140 unique sequences.
## Sample 49 - 49167 reads in 12886 unique sequences.
## Sample 50 - 124398 reads in 31112 unique sequences.
## Sample 51 - 123435 reads in 35787 unique sequences.
## Sample 52 - 122533 reads in 34373 unique sequences.
```

```r
dadaRs = dada(filtRs, errR, multithread = TRUE)
```

```
## Sample 1 - 50997 reads in 14119 unique sequences.
## Sample 2 - 129948 reads in 31988 unique sequences.
## Sample 3 - 133768 reads in 30841 unique sequences.
## Sample 4 - 129102 reads in 27720 unique sequences.
## Sample 5 - 121156 reads in 27901 unique sequences.
## Sample 6 - 133796 reads in 30429 unique sequences.
## Sample 7 - 122418 reads in 34611 unique sequences.
## Sample 8 - 120891 reads in 34339 unique sequences.
## Sample 9 - 72059 reads in 20203 unique sequences.
```

```
## Sample 10 - 138862 reads in 42325 unique sequences.
## Sample 11 - 136191 reads in 40507 unique sequences.
## Sample 12 - 132129 reads in 40667 unique sequences.
## Sample 13 - 130915 reads in 41167 unique sequences.
## Sample 14 - 122136 reads in 39042 unique sequences.
## Sample 15 - 123426 reads in 35887 unique sequences.
## Sample 16 - 130415 reads in 39222 unique sequences.
## Sample 17 - 132945 reads in 38542 unique sequences.
## Sample 18 - 131107 reads in 39954 unique sequences.
## Sample 19 - 99749 reads in 23670 unique sequences.
## Sample 20 - 132778 reads in 38658 unique sequences.
## Sample 21 - 130653 reads in 38524 unique sequences.
## Sample 22 - 134573 reads in 37765 unique sequences.
## Sample 23 - 126227 reads in 37957 unique sequences.
## Sample 24 - 138808 reads in 38159 unique sequences.
## Sample 25 - 123638 reads in 37255 unique sequences.
## Sample 26 - 133383 reads in 39942 unique sequences.
## Sample 27 - 127008 reads in 40331 unique sequences.
## Sample 28 - 130717 reads in 39862 unique sequences.
## Sample 29 - 126983 reads in 38403 unique sequences.
## Sample 30 - 125192 reads in 28552 unique sequences.
## Sample 31 - 125192 reads in 36897 unique sequences.
## Sample 32 - 123257 reads in 36870 unique sequences.
## Sample 33 - 136461 reads in 38455 unique sequences.
## Sample 34 - 122183 reads in 35046 unique sequences.
## Sample 35 - 135949 reads in 41043 unique sequences.
## Sample 36 - 130411 reads in 40089 unique sequences.
## Sample 37 - 138592 reads in 40482 unique sequences.
## Sample 38 - 134629 reads in 41835 unique sequences.
## Sample 39 - 129737 reads in 32420 unique sequences.
## Sample 40 - 127445 reads in 39127 unique sequences.
## Sample 41 - 123235 reads in 41139 unique sequences.
## Sample 42 - 126827 reads in 40371 unique sequences.
## Sample 43 - 129972 reads in 39615 unique sequences.
## Sample 44 - 138434 reads in 40112 unique sequences.
## Sample 45 - 121764 reads in 38010 unique sequences.
## Sample 46 - 125875 reads in 37637 unique sequences.
## Sample 47 - 135450 reads in 34023 unique sequences.
## Sample 48 - 121771 reads in 36164 unique sequences.
## Sample 49 - 49167 reads in 11354 unique sequences.
## Sample 50 - 124398 reads in 27768 unique sequences.
## Sample 51 - 123435 reads in 32154 unique sequences.
## Sample 52 - 122533 reads in 30817 unique sequences.
```

```r
mergers = mergePairs(dadaF = dadaFs,
                     derepF = filtFs,
                     dadaR = dadaRs,
                     derepR = filtRs)

mergers = mergePairs(dadaF = dadaFs,
                     derepF = filtFs,
                     dadaR = dadaRs,
                     derepR = filtRs,
                     verbose = TRUE)
```

```
## 45401 paired-reads (in 770 unique pairings) successfully merged out of 47957 (in 1678 pairings) inpu
## 116654 paired-reads (in 1105 unique pairings) successfully merged out of 123677 (in 3627 pairings) in
## 121747 paired-reads (in 1044 unique pairings) successfully merged out of 128417 (in 3371 pairings) in
## 118589 paired-reads (in 1109 unique pairings) successfully merged out of 123742 (in 3135 pairings) in
## 110974 paired-reads (in 989 unique pairings) successfully merged out of 116117 (in 2913 pairings) inp
## 121768 paired-reads (in 1140 unique pairings) successfully merged out of 128083 (in 3605 pairings) in
## 108395 paired-reads (in 1288 unique pairings) successfully merged out of 114952 (in 4180 pairings) in
## 107749 paired-reads (in 1385 unique pairings) successfully merged out of 113585 (in 3811 pairings) in
## 64637 paired-reads (in 1017 unique pairings) successfully merged out of 67742 (in 2459 pairings) inpu
## 122143 paired-reads (in 1558 unique pairings) successfully merged out of 129914 (in 4781 pairings) in
## 119203 paired-reads (in 1590 unique pairings) successfully merged out of 127530 (in 4765 pairings) in
## 116329 paired-reads (in 1580 unique pairings) successfully merged out of 123845 (in 4499 pairings) in
## 115097 paired-reads (in 1470 unique pairings) successfully merged out of 122541 (in 4517 pairings) in
## 107445 paired-reads (in 1505 unique pairings) successfully merged out of 113808 (in 4193 pairings) in
## 110510 paired-reads (in 1425 unique pairings) successfully merged out of 115964 (in 3843 pairings) in
## 115972 paired-reads (in 1440 unique pairings) successfully merged out of 122567 (in 4222 pairings) in
## 118201 paired-reads (in 1537 unique pairings) successfully merged out of 124883 (in 4323 pairings) in
## 116513 paired-reads (in 1510 unique pairings) successfully merged out of 123049 (in 4330 pairings) in
## 91062 paired-reads (in 1118 unique pairings) successfully merged out of 95076 (in 2844 pairings) inpu
## 118260 paired-reads (in 1601 unique pairings) successfully merged out of 124668 (in 4367 pairings) in
## 115536 paired-reads (in 1504 unique pairings) successfully merged out of 122581 (in 4379 pairings) in
## 120690 paired-reads (in 1585 unique pairings) successfully merged out of 126598 (in 4352 pairings) in
## 112896 paired-reads (in 1495 unique pairings) successfully merged out of 118788 (in 4012 pairings) in
## 123916 paired-reads (in 1502 unique pairings) successfully merged out of 130894 (in 4173 pairings) in
## 109842 paired-reads (in 1504 unique pairings) successfully merged out of 115920 (in 3998 pairings) in
## 119070 paired-reads (in 1462 unique pairings) successfully merged out of 125889 (in 4192 pairings) in
## 112121 paired-reads (in 1619 unique pairings) successfully merged out of 118536 (in 4381 pairings) in
## 116501 paired-reads (in 1528 unique pairings) successfully merged out of 122688 (in 4118 pairings) in
## 113367 paired-reads (in 1426 unique pairings) successfully merged out of 119378 (in 4075 pairings) in
## 113907 paired-reads (in 1038 unique pairings) successfully merged out of 119983 (in 3189 pairings) in
## 112019 paired-reads (in 1548 unique pairings) successfully merged out of 117698 (in 4082 pairings) in
## 110555 paired-reads (in 1416 unique pairings) successfully merged out of 115956 (in 3804 pairings) in
## 123050 paired-reads (in 1665 unique pairings) successfully merged out of 128671 (in 4223 pairings) in
## 109858 paired-reads (in 1445 unique pairings) successfully merged out of 115148 (in 3656 pairings) in
## 120981 paired-reads (in 1643 unique pairings) successfully merged out of 127092 (in 4511 pairings) in
## 114823 paired-reads (in 1515 unique pairings) successfully merged out of 121841 (in 4373 pairings) in
```

```
## 122711 paired-reads (in 1560 unique pairings) successfully merged out of 129880 (in 4542 pairings) i
## 118336 paired-reads (in 1572 unique pairings) successfully merged out of 125713 (in 4562 pairings) i
## 116905 paired-reads (in 1105 unique pairings) successfully merged out of 123728 (in 3589 pairings) i
## 112858 paired-reads (in 1373 unique pairings) successfully merged out of 119534 (in 3955 pairings) i
## 107610 paired-reads (in 1601 unique pairings) successfully merged out of 114635 (in 4489 pairings) i
## 112019 paired-reads (in 1494 unique pairings) successfully merged out of 118342 (in 4071 pairings) i
## 115353 paired-reads (in 1405 unique pairings) successfully merged out of 122057 (in 4084 pairings) i
## 123594 paired-reads (in 1609 unique pairings) successfully merged out of 129728 (in 4401 pairings) i
## 107685 paired-reads (in 1465 unique pairings) successfully merged out of 113999 (in 4030 pairings) i
## 111927 paired-reads (in 1545 unique pairings) successfully merged out of 117629 (in 4139 pairings) i
## 120898 paired-reads (in 1286 unique pairings) successfully merged out of 128985 (in 4349 pairings) i
## 108319 paired-reads (in 1495 unique pairings) successfully merged out of 114108 (in 4105 pairings) i
## 45293 paired-reads (in 507 unique pairings) successfully merged out of 46921 (in 1160 pairings) inpu
## 113457 paired-reads (in 1004 unique pairings) successfully merged out of 119565 (in 3129 pairings) i
## 111437 paired-reads (in 1084 unique pairings) successfully merged out of 117818 (in 3520 pairings) i
## 111419 paired-reads (in 1032 unique pairings) successfully merged out of 117316 (in 3218 pairings) i
```

```
seqtab = makeSequenceTable(mergers)

seqtab.nochim = removeBimeraDenovo(seqtab,
                    method = "consensus",
                    multithread = TRUE,
                    verbose = TRUE)
```

```
## Identified 12505 bimeras out of 24457 input sequences.
```

```
taxa = assignTaxonomy(seqtab.nochim,
                      "/home/user/qiime/tax_datab/silva_nr99_v138_train_set.fa.gz",
                      multithread = TRUE,
                      tryRC = TRUE)

taxa_spec = addSpecies(taxa, "/home/user/qiime/tax_datab/primate_consensus_sequences.fasta")
```

```
dim(seqtab.nochim)
```

```
## [1]    52 11952
```

```
getN <- function(x) sum(getUniques(x))
track <- cbind(out, sapply(dadaFs, getN), sapply(dadaRs, getN), sapply(mergers, getN), rowSums(seqtab.n
# If processing a single sample, remove the sapply calls: e.g. replace sapply(dadaFs, getN) with getN(d
colnames(track) <- c("input", "filtered", "denoisedF", "denoisedR", "merged", "nonchim")
rownames(track) <- sample.names
track
```

```
##       input filtered denoisedF denoisedR merged nonchim
## PR1   54390    50997     48904     48871  45401   43053
## PR10 139585   129948    125831    125809 116654  106324
## PR11 144022   133768    130179    130189 121747  109957
## PR15 140119   129102    125615    125460 118589  111736
```

```
## PR16 131087   121156    117703    117913 110974  103019
## PR17 143767   133796    129910    130076 121768  111197
## PR18 131629   122418    117387    117438 108395  100935
## PR19 130907   120891    116035    115961 107749  101180
## PR2   76996    72059     69274     68919  64637   60694
## PR20 149595   138862    132847    132943 122143  115554
## PR21 146643   136191    130278    130486 119203  112972
## PR22 142494   132129    126625    126499 116329  111335
## PR23 141901   130915    125272    125418 115097  108830
## PR24 132194   122136    116582    116498 107445  102231
## PR26 132451   123426    118498    118210 110510  106079
## PR27 141481   130415    125276    125007 115972  109892
## PR28 143010   132945    127757    127370 118201  112542
## PR29 141497   131107    125843    125542 116513  111298
## PR3  106331    99749     96706     96509  91062   84233
## PR30 143357   132778    127356    127224 118260  112187
## PR31 141169   130653    125325    125150 115536  109057
## PR32 144153   134573    129251    128937 120690  116072
## PR33 135766   126227    121198    121140 112896  108484
## PR34 148637   138808    133604    133445 123916  120057
## PR35 133039   123638    118508    118469 109842  106533
## PR36 143941   133383    128532    128077 119070  114629
## PR37 136394   127008    121268    121266 112121  108886
## PR38 140797   130717    125349    125246 116501  113115
## PR39 136808   126983    121838    121733 113367  109793
## PR4  134624   125192    121819    121723 113907  103371
## PR40 134122   125192    120217    120001 112019  108879
## PR41 132808   123257    118482    118177 110555  107790
## PR42 146347   136461    131188    130975 123050  119243
## PR43 131536   122183    117502    117332 109858  106236
## PR44 147128   135949    130205    129747 120981  117564
## PR45 140728   130411    124614    124602 114823  110757
## PR46 149805   138592    132693    132659 122711  118474
## PR47 145227   134629    128667    128515 118336  115439
## PR5  139679   129737    125675    125846 116905  106020
## PR51 138574   127445    122286    121983 112858  109236
## PR52 133875   123235    117513    117263 107610  104500
## PR55 137548   126827    121306    120940 112019  109167
## PR56 141078   129972    124714    124468 115353  111928
## PR57 148900   138434    132627    132445 123594  120721
## PR58 132154   121764    116646    116475 107685  105137
## PR59 136298   125875    120607    119984 111927  108899
## PR6  145100   135450    131186    131116 120898  108510
## PR60 131368   121771    116735    116390 108319  105332
## PR61  52366    49167     47625     47599  45293   42134
## PR7  133280   124398    121088    121275 113457  104223
## PR8  133268   123435    119668    119767 111437  100745
## PR9  132519   122533    118976    119119 111419  102119
```

```r
dim(taxa_spec)
```

```
## [1] 11952     7
```

```r
write.csv(taxa_spec, "dada2_taxonomy.csv")
write.csv(seqtab.nochim, "dada2_feature_table_nochim.csv")
```

```r
bifidos = c()

my_range = 1:dim(taxa_spec)[1]
for (i in my_range){
  if (match(taxa_spec[i, 6], "Bifidobacterium", nomatch = 0)){
    print(taxa_spec[i, 6:7])
    bifidos = c(bifidos, rownames(taxa_spec)[i])
  }
}
```

```
##               Genus             Species
##   "Bifidobacterium" "callitrichos_N036"
##             Genus           Species
## "Bifidobacterium"          "parmae"
##             Genus           Species
## "Bifidobacterium"                NA
##             Genus           Species
## "Bifidobacterium"                NA
##                Genus               Species
##    "Bifidobacterium" "vansinderenii_N113"
##             Genus           Species
## "Bifidobacterium"       "sp._N055"
##             Genus           Species
## "Bifidobacterium"                NA
##             Genus           Species
## "Bifidobacterium"                NA
##             Genus           Species
## "Bifidobacterium"       "sp._N097"
##             Genus           Species
## "Bifidobacterium"                NA
##             Genus           Species
## "Bifidobacterium"                NA
##             Genus           Species
## "Bifidobacterium"                NA
##             Genus           Species
## "Bifidobacterium"                NA
##             Genus           Species
## "Bifidobacterium"                NA
##             Genus           Species
## "Bifidobacterium"   "goeldii_N002"
##             Genus           Species
## "Bifidobacterium"                NA
##             Genus           Species
## "Bifidobacterium"                NA
##             Genus           Species
## "Bifidobacterium"   "ramosum_N042"
##             Genus           Species
## "Bifidobacterium"  "tissieri_N043"
##             Genus           Species
## "Bifidobacterium"                NA
##             Genus           Species
## "Bifidobacterium"                NA
##             Genus           Species
## "Bifidobacterium"                NA
```

```
##             Genus           Species
## "Bifidobacterium"                NA
##             Genus           Species
## "Bifidobacterium"                NA
##             Genus           Species
## "Bifidobacterium"                NA
##             Genus           Species
## "Bifidobacterium"                NA
##             Genus           Species
## "Bifidobacterium"                NA
##             Genus           Species
## "Bifidobacterium"                NA
##                         Genus                        Species
##             "Bifidobacterium" "animalis_subsp._lactis_N045"
##             Genus           Species
## "Bifidobacterium"                NA
##             Genus           Species
## "Bifidobacterium"                NA
##             Genus           Species
## "Bifidobacterium"                NA
##             Genus           Species
## "Bifidobacterium"                NA
##             Genus           Species
## "Bifidobacterium"                NA
##             Genus           Species
## "Bifidobacterium"                NA
##             Genus           Species
## "Bifidobacterium"                NA
##             Genus           Species
## "Bifidobacterium"                NA
##             Genus           Species
## "Bifidobacterium"                NA
##             Genus           Species
## "Bifidobacterium"                NA
##             Genus           Species
## "Bifidobacterium"                NA
##             Genus           Species
## "Bifidobacterium"                NA
##             Genus           Species
## "Bifidobacterium"                NA
##             Genus           Species
## "Bifidobacterium"                NA
##             Genus           Species
## "Bifidobacterium"                NA
##             Genus           Species
## "Bifidobacterium"                NA
##             Genus           Species
## "Bifidobacterium"                NA
##             Genus           Species
## "Bifidobacterium"                NA
##             Genus           Species
## "Bifidobacterium"                NA
```

```
##              Genus           Species
## "Bifidobacterium"                NA
##              Genus           Species
## "Bifidobacterium"                NA
##              Genus           Species
## "Bifidobacterium"                NA
```

```r
write.csv(bifidos, "bifidos.csv")
```

Supplementary S4

March 25, 2021

1 Sequence analysis

[1]:
```
import sys
import copy

sys.path.append("/home/user/Programs/seqDataClassCluster")

import pandas as pd
import numpy as np
import random

import plotly.express as px
import plotly.graph_objs as go
from plotly.subplots import make_subplots

from seqDataClass import seqObject
#import seqDataClass

template = 'plotly_white'
```

[2]:
```
filepath = "/home/user/qiime/monkeys/"
```

[3]:
```
seq1 = seqObject(mappingFile = filepath+"mapping file_monkeys_simplified.csv",
                 taxonomyFile = filepath+"dada2_taxonomy_updated.csv",
                 featureFile = filepath+"dada2_feature_table_nochim.csv",
                 mappingSep = ";",
                 taxonomySep = ",",
                 featureSep = ",",
                 sampleNamesColumn = "sample-id",
                 #featureFormat = "dada2",
                 #taxonomyFormat = "dada2"
                 )
```

```
Initializing data loading.
Loading mapping file
Loading taxonomy
Recognizing a DADA2 format.
```

```
Matching features between feature file and taxonomy file.
Features match.
```

[4]:
```
seq1.extract_rep_seq(filename=filepath+"rep_seq.fasta", renameFeatures=True)
```

1.1 Checking the Bifidobacteria species assignment

[5]:
```
tax_dict = {}
with open("rep_seq.fasta", 'r') as f_in:
    for line in f_in:
        if '>' in line:
            name = line[1:].rstrip()
        else:
            sequence = line
            tax_dict[name] = sequence.rstrip()

df_ind = seq1.data.xs("Bifidobacterium", level="Genus", axis=0).index.
 ↪to_frame(index=False)
with open("bifidos_rep_seq.fasta", 'w') as f_out:
    for feature, species in zip(df_ind["feature-id"], df_ind["Species"]):
        #print(f"{feature}, Bifidobacterium {species} : {tax_dict[feature]}")
        f_out.write(">" + feature + " Bifidobacterium " + species + "\n")
        f_out.write(tax_dict[feature] + "\n")
```

[6]:
```
%%bash
head -n 6 bifidos_rep_seq.fasta
```

```
>feature0 Bifidobacterium callitrichos
TAATACGTAGGGTGCAAGCGTTATCCGGAATTATTGGGCGTAAAGAGCTCGTAGGCGGTTCGTCGCGTCTGGTGTGAAAG
TCCATCGCTTAACGGTGGATCTGCGCCGGGTACGGGCGGGCTGGAGTGCGGTAGGGGAGACTGGAATTCCCGGTGTAACG
GTGGAATGTGTAGATATCGGGAAGAACACCAATGGCGAAGGCAGGTCTCTGGGCCGTTACTGACGCTGAGGAGCGAAAGC
GTGGGGAGCGAACAGGATTA
>feature1 Bifidobacterium parmae 1
TAATACGTAGGGTGCAAGCGTTATCCGGAATTATTGGGCGTAAAGGGCTCGTAGGCGGTTCGTCGCGTCCGGTGTGAAAG
CCCATCGCTTAACGGTGGGTCTGCGCCGGGTACGGGCGGGCTGGAGTGCGGTAGGGGAGACTGGAATTCCCGGTGTAACG
GTGGAATGTGTAGATATCGGGAGGAACACCAATGGCGAAGGCAGGTCTCTGGGCCGTTACTGACGCTGAGGAGCGAAAGC
GTGGGGAGCGAACAGGATTA
>feature19 Bifidobacterium saguini
TAATACGTAGGGCGCAAGCGTTATCCGGAATTATTGGGCGTAAAGGGCTCGTAGGCGGTTCGTCGCGTCCGGTGTGAAAG
TCCATCGCTTAACGGTGGATCCGCGCCGGGTACGGGCGGGCTTGAGTGCGGTAGGGGAGACTGGAATTCCCGGTGTAACG
GTGGAATGTGTAGATATCGGGAAGAACACCAATGGCGAAGGCAGGTCTCTGGGCCGTTACTGACGCTGAGGAGCGAAAGC
GTGGGGAGCGAACAGGATTA
```

[7]:
```
%%bash
/home/user/Programs/anaconda3/envs/qiime2/bin/blastn -query bifidos_rep_seq.
 ↪fasta \
```

```
                                                     -db ../tax_datab/
↪primate_reference/primate_consensus_sequences_all.fasta \
                                                     -outfmt 1 \
                                                     -num_alignments 10 \
                                                     -num_descriptions 1 \
                                                     -out bifido_alignment.txt \
                                                     -dust no \
                                                     -task blastn
```

[8]:
```
%%bash
#more bifido_alignment.txt
head -n 45 bifido_alignment.txt
```

```
BLASTN 2.9.0+


Reference: Stephen F. Altschul, Thomas L. Madden, Alejandro A.
Schaffer, Jinghui Zhang, Zheng Zhang, Webb Miller, and David J.
Lipman (1997), "Gapped BLAST and PSI-BLAST: a new generation of
protein database search programs", Nucleic Acids Res. 25:3389-3402.



Database: primate_consensus_sequences_all.fasta
           61 sequences; 87,095 total letters



Query= feature0 Bifidobacterium callitrichos

Length=260
                                                                      Score
E
Sequences producing significant alignments:                          (Bits)
Value

N099_B.callitrichos_99.50% 16S ribosomal RNA gene, partial sequence   470
6e-135



Query_1                     1
TAATACGTAGGGTGCAAGCGTTATCCGGAATTATTGGGCGTAAAGAGCTCGTAGGCGGTT  60
N099_B.callitrichos_99.50%  448
…  507
N058_B.callitrichos_99.15%  452
…  511
```

```
N037_B.callitrichos_98.86%  445
…  504
N036_B.callitrichos_99.57%  448
…  507
N035_B.callitrichos_99.08%  447
…  506
NR_025452.1_B.scardovii     480
…G…  539
N098_B.angulatum_99.36%     452
…  511
N061_B.angulatum_99.37%     455
…  514
N007_B.angulatum_99.72%     451
…  510
N002_B.goeldii_99.58%       452
…  511

Query_1                     61
CGTCGCGTCTGGTGTGAAAGTCCATCGCTTAACGGTGGATCTGCGCCGGGTACGGGCGGG  120
N099_B.callitrichos_99.50%  508
…  567
N058_B.callitrichos_99.15%  512
…  571
N037_B.callitrichos_98.86%  505
…  564
N036_B.callitrichos_99.57%  508
…  567
N035_B.callitrichos_99.08%  507
…  566
NR_025452.1_B.scardovii     540
…  599
N098_B.angulatum_99.36%     512
…C…  571
```

1.2 Data normalization : Rarefaction to even depth

[9]:
```
#seq1.data.columns.to_frame(index = False).iloc[:, 2:]
sample_sums = seq1.data.sum(axis=1, level="sample-id").sum()
sample_sums.name = "value"
sample_sums = sample_sums.to_frame()
sample_sums["ID"] = sample_sums.index

import plotly.graph_objects as go
fig = go.Figure(
    data=[
        go.Bar(
```

```
            y=list(sample_sums["value"]),
            x=list(sample_sums["ID"])
               )
          ],
    layout_title_text="Number of sequences per sample",
)
#fig.update_layout(hovermode='y')
fig.update_layout(template=template)
fig.show()
```

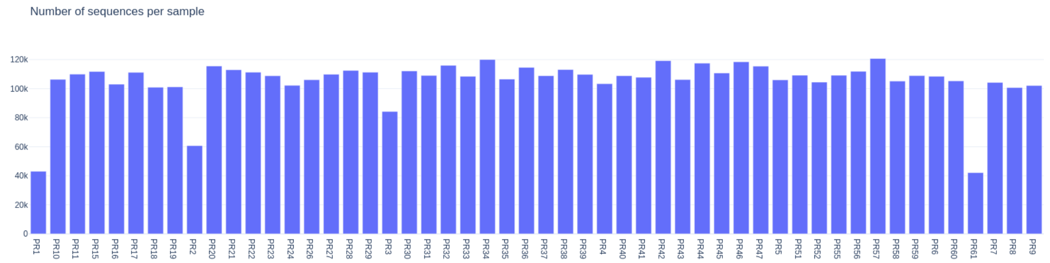

Dataset was normalized to the lowest sample count, which is sample **PR61** with 42 134 sequences.

[10]:
```
seq1.rarefy_to_even_depth(seqDepth=42134, seed=123)
seq1.
 ↪extract_features(tax_level="feature-id",file_name="monkeys_otu_table_normalized.
 ↪csv")
```

```
Removing 2513 features/OTUs that no longer appear in any sample.
```

[10]:
```
sample-id      PR1  PR10  PR11   PR15  PR16  PR17  PR18  PR19   PR2  PR20  \
feature-id
feature0      1577  3831  3820   1722  3188  3392  1732  1062  1699   670
feature1       472  5284  5531   1035  2800  4237  1193   936   715   591
feature2        97  2080  1865   1142  1361  2715  1127   204   327   360
feature3      4770    83    59    186   441   232  1064  1023  2554  1222
feature4       288   803  1425  14955  5788  2055   637   110    94    12
…             …    …    …    …    …    …    …    …    …    …
feature11941     0     0     0      0     0     0     0     0     0     0
feature11942     0     0     0      0     0     0     0     0     0     0
feature11946     0     0     0      0     0     0     0     0     0     0
feature11947     0     0     0      0     0     0     0     0     0     0
feature11949     0     0     0      0     0     0     0     0     0     0

sample-id      …  PR56  PR57  PR58  PR59   PR6  PR60  PR61   PR7   PR8   PR9
feature-id     …
feature0       …   116    60   161   103  2543    66   628  3469  1993  4764
```

```
feature1      …   220   17    89   133  2220    10  2211  2626  1277  4694
feature2      …   183   10   180   411  4181     8   187  4909  2857  1866
feature3      …   145  359  1048   523   194  1629  2001    91   282   160
feature4      …    45  671   199    58   437     2   138   687   873  1300
…           …   …   …   …   …   …   …   …   …   …   …
feature11941  …     0    0     0     0     0     0     0     0     0     1
feature11942  …     0    0     0     0     0     0     0     0     0     2
feature11946  …     0    0     0     0     0     0     0     0     0     1
feature11947  …     0    0     0     0     0     0     0     0     0     3
feature11949  …     0    0     0     0     0     0     0     0     0     2

[9439 rows x 52 columns]
```

1.3 Relative proportion of the bacterial community

Following is a relative bacterial abundance in the normalized samples.

1.3.1 Relative abundance plots sub-order

Phylum level

[11]:
```python
fig = seq1.stacked_barplot(tax_level="Phylum",
                           map_level="sub-order",
                           plotter="plotly", treshold_mean=0.01)

fig.update_layout(title=dict(text='Relative abundance sub-order: Phylum level'),
                  width=600,
                  height=600,
                  barmode="relative",
                  template=template
                 )

fig.write_image("final_files/relative_adundance_sub-order_phylum.pdf")

fig.show()
```

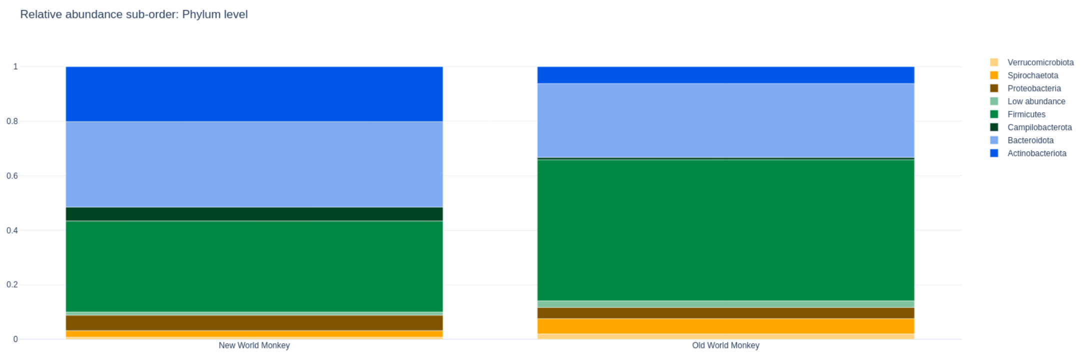

[12]:

```
fig = seq1.stacked_barplot(tax_level="Phylum",
                           map_level=["sub-order", "sample-id"],
                           plotter="plotly", treshold_mean=0.01)

fig.update_layout(title=dict(text='Relative abundance per sample sub-order:␣
 ↪Phylum level'),
                  width=1300,
                  height=600,
                  barmode="relative",
                  template=template
                 )

fig.show()
```

end

/home/user/Programs/seqDataClassCluster/seqDataClass.py:1017:
SettingWithCopyWarning:

A value is trying to be set on a copy of a slice from a DataFrame

See the caveats in the documentation: https://pandas.pydata.org/pandas-docs/stable/user_guide/indexing.html#returning-a-view-versus-a-copy

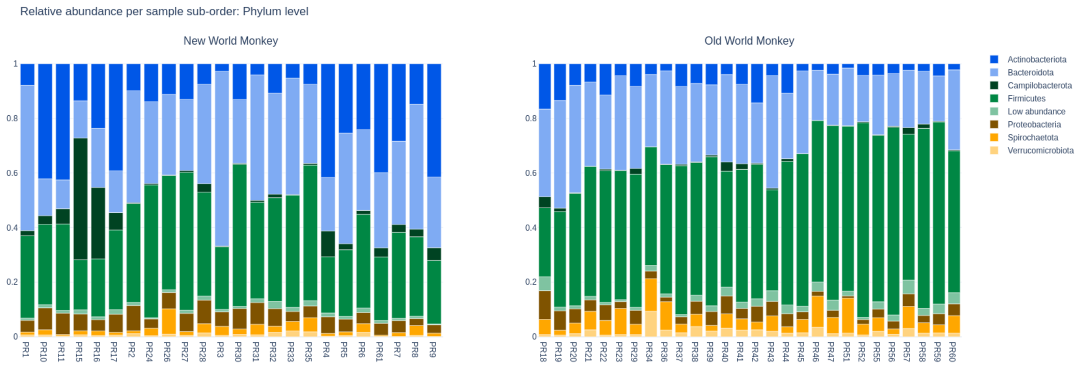

Family level

[13]:
```
fig = seq1.stacked_barplot(tax_level="Family",
                           map_level="sub-order",
                           plotter="plotly", treshold_mean=0.015)

fig.update_layout(title=dict(text='Relative abundance feed classification:␣
 ↪Family level'),
                  width=600,
                  height=600,
                  barmode="relative",
                  template=template
                 )

fig.write_image("final_files/relative_adundance_sub-order_family.pdf")

fig.show()
```

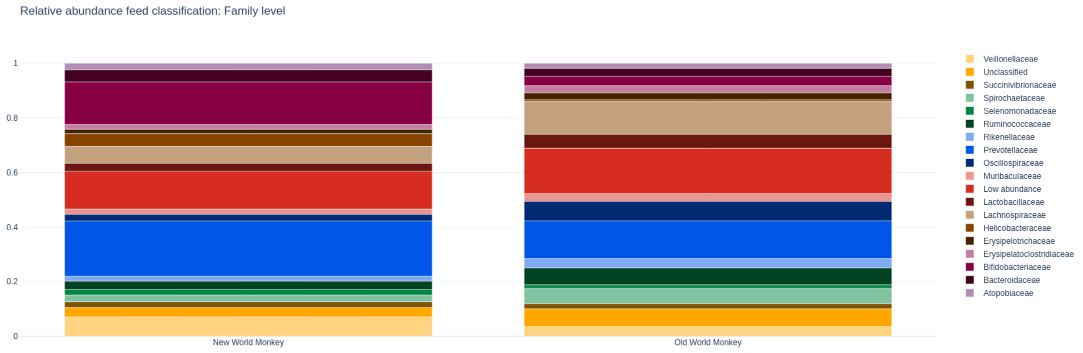

[14]:
```
fig = seq1.stacked_barplot(tax_level="Family",
                           map_level=["sub-order", "sample-id"],
                           plotter="plotly", treshold_mean=0.015)

fig.update_layout(title=dict(text='Relative abundance sub-order: Family level'),
                  width=1300,
                  height=600,
                  barmode="relative",
                  template=template
                 )


fig.show()
```

end

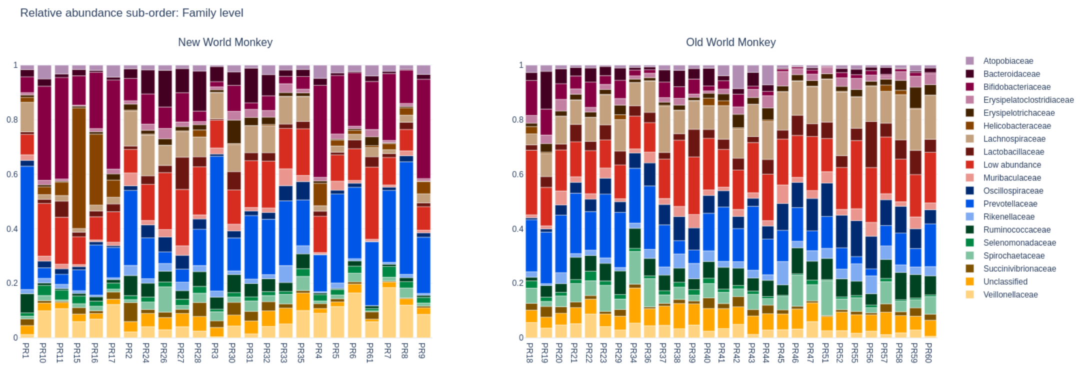

1.3.2 Relative abundance plots diet

Phylum level

[15]:
```
fig = seq1.stacked_barplot(tax_level="Phylum",
                           map_level="feed",
                           plotter="plotly", treshold_mean=0.01)

fig.update_layout(title=dict(text='Relative abundance feed classification:␣
 ↪Phylum level'),
                  width=600,
                  height=600,
                  barmode="relative",
                  template=template
                 )
```

```
fig.write_image("final_files/relative_adundance_feed_phylum.pdf")

fig.show()
```

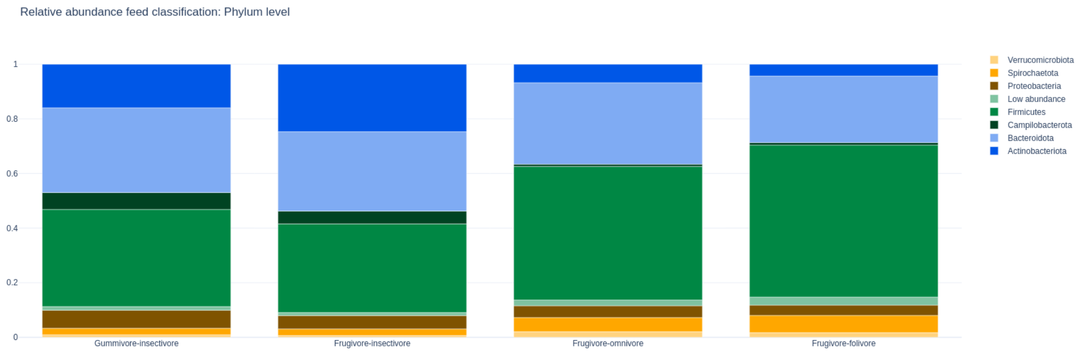

Family level

[16]:
```
fig = seq1.stacked_barplot(tax_level="Family",
                           map_level="feed",
                           plotter="plotly", treshold_mean=0.015)

fig.update_layout(title=dict(text='Relative abundance feed classification:␣
 ↪Family level'),
                  width=600,
                  height=600,
                  barmode="relative",
                  template=template
                 )

fig.write_image("final_files/relative_adundance_feed_family.pdf")

fig.show()
```

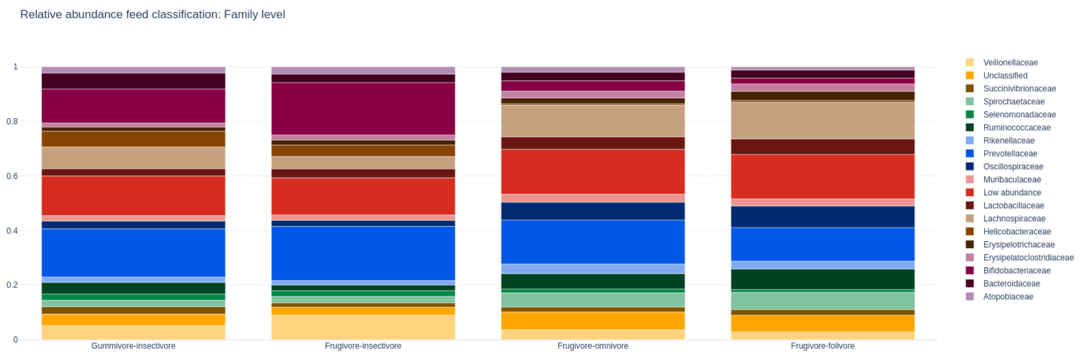
Relative abundance feed classification: Family level
1
0.8
0.6
0.4
0.2
0
Gummivore-insectivore
Frugivore-insectivore
Frugivore-omnivore
Frugivore-folivore
Veillonellaceae
Unclassified
Succinivibrionaceae
Spirochaetaceae
Selenomonadaceae
Ruminococcaceae
Rikenellaceae
Prevotellaceae
Oscillospiraceae
Muribaculaceae
Low abundance
Lactobacillaceae
Lachnospiraceae
Helicobacteraceae
Erysipelotrichaceae
Erysipelatoclostridiaceae
Bifidobacteriaceae
Bacteroidaceae
Atopobiaceae

Supplementary S5

Table I: Cultivation bacterial counts after microbiological analysis of primate faecal samples

	Frugivore-folivore	Frugivore-omnivore	Frugivore-insectivore	Gummivore-insectivore
**WSP**	8.72±0.49 [ab]	8.60±0.78 [b]	9.46±0.57 [a]	9.63±0.71 [a]
**MUP**	6.58±1.05 [b]	7.07±1.01 [b]	9.19±0.96 [a]	8.99±1.19 [a]
**NORF**	4.29±1.95 [b]	4.22±2.13 [b]	9.15±0.76 [a]	8.46±2.34 [a]

Counts of bacteria are averages in log CFU g^{-1} ± standard deviation per primate feed category – frugivore-folivore (n=8), frugivore-omnivore (n=21), frugivore-insectivore (n=13), and gummivore-insectivore (n=10). Superscript letters represent statistical differences ($\alpha = 0.05$) evaluated by analysis of variance with one-way ANOVA (Scheffe's test) using STATISTICA software (StatSoft, Prague, Czechia). WSP, medium for total counts of anaerobic bacteria; MUP, selective medium with mupirocin and acetic acid; NORF, selective medium with norfloxacin, mupirocin, and acetic acid.

Table II: P-values in one-way ANOVA (Scheffe's test) within the WSP

	Gummivore-insectivore	Frugivore-omnivore	Frugivore-insectivore	Frugivore-folivore
**Gummivore-insectivore**		4.04e-03	0.95	0.06
**Frugivore-omnivore**	4.04e-03		1.03e-02	0.98
**Frugivore-insectivore**	0.95	1.03e-02		0.14
**Frugivore-folivore**	0.06	0.98	0.14	

Table III: P-values in one-way ANOVA (Scheffe's test) within the MUP

	Gummivore-insectivore	Frugivore-omnivore	Frugivore-insectivore	Frugivore-folivore
**Gummivore-insectivore**		2.61e-04	0.98	2.03e-04
**Frugivore-omnivore**	2.61e-04		1.19e-05	0.73
**Frugivore-insectivore**	0.98	1.19e-05		2.22e-05
**Frugivore-folivore**	2.03e-04	0.73	2.22e-05	

Table IV: P-values in one-way ANOVA (Scheffe's test) within the NORF

	Gummivore-insectivore	Frugivore-omnivore	Frugivore-insectivore	Frugivore-folivore
**Gummivore-insectivore**		1.06e-05	0.86	0.47e-03
**Frugivore-omnivore**	1.06e-05		5.79e-08	1.00
**Frugivore-insectivore**	0.86	5.79e-08		1.57e-05
**Frugivore-folivore**	0.47e-03	1.00	1.57e-05	
